# Comparative efficacy of non-invasive brain stimulation for attention-deficit/hyperactivity disorder: a systematic review and network meta-analysis

**DOI:** 10.3389/fneur.2025.1650154

**Published:** 2025-10-02

**Authors:** Xinwen Liang, Xiaoyu Wei, Yan Huang, Jing Li, Huan Feng, Jingyuan Fan, Longguo Zhang, Zhijiang Wang, Xin Zhao, Weimin Pan, Rui Liu

**Affiliations:** ^1^Department of Rehabilitation Medicine, Tangdu Hospital, Fourth Military Medical University, Xi'an, China; ^2^Xi'an Gaoxin Hospital, Xi'an, China; ^3^Engineering Research Center of Innovative Technology of Intelligent Sports Equipment, Universities of Shaanxi Province, Xi'an, China; ^4^Xi'an Physical Education University, Xi'an, China

**Keywords:** NIBS, ADHD, cognitive functions, core symptoms, network meta-analysis

## Abstract

**Introduction:**

In recent years, non-invasive brain stimulation (NIBS) interventions for attention-deficit/hyperactivity disorder (ADHD) have received increasing attention. However, which of the various NIBS methods is more effective in improving cognitive functions and core symptoms in patients with ADHD remains unclear.

**Methods:**

Randomized controlled trials (RCTs) on NIBS in patients with ADHD were searched. Standardized mean differences (SMDs) for cognitive functions and core symptoms changes were pooled in Bayesian network meta-analyses.

**Result:**

After reviewing 3,976 retrieved citations, a total of 37 RCTs (*N* = 1,615 participants) were included. This NMA provides evidence that none of the NIBS interventions significantly improved inhibitory control compared to sham controls. However, a statistically significant difference was observed between anodal transcranial direct current stimulation (tDCS) over the left DLPFC plus cathodal tDCS over the right supraorbital area 1.5 mA (SMD = −0.87, 95% CI: −1.80 to −0.07) and High-definition anodal transcranial direct current stimulation over the vertex 0.25 mA (SMD = −1.04, 95% CI: −2.09 to 0.00). In terms of working memory, anodal tDCS over the left DLPFC plus cathodal tDCS over the right DLPFC (SMD = 0.95, 95% CI: 0.05–1.84) and anodal tDCS over the right inferior frontal cortex (rIFC) plus cathodal tDCS over the right supraorbital area (SMD = 0.86, 95% CI: 0.28–1.45) were associated with significant improvements compared to sham stimulation. For cognitive flexibility, only anodal tDCS over the left DLPFC plus cathodal tDCS over the right supraorbital area (SMD = −0.76, 95% CI: −1.31 to −0.21) demonstrated a statistically significant benefit relative to sham. Regarding inattention, both transcranial pulse stimulation (SMD = −2.62, 95% CI: −6.35 to 1.12) and transcranial alternating current stimulation 10 Hz (SMD = −2.35, 95% CI: −5.00 to 0.30) showed favorable trends in comparison with sham; however, these differences did not reach statistical significance, though they approached the threshold. Finally, no NIBS intervention was found to significantly improve hypersensitivity or impulsivity when compared to sham stimulation.

**Conclusion:**

The dual-tDCS and a-tDCS may be considered among the preferred NIBS interventions for improving cognitive function in ADHD. Specifically, anodal tDCS over the left DLPFC plus cathodal tDCS over the right supraorbital area improved cognitive flexibility; while anodal tDCS over the left DLPFC plus cathodal tDCS over the right DLPFC enhanced working memory; both dual-tDCS and a-tDCS demonstrated superior efficacy relative to repetitive transcranial magnetic stimulation (rTMS) for inhibitory control; further research is needed to investigate TPS for improving attention and impulsivity.

## 1 Introduction

Attention-deficit/hyperactivity disorder (ADHD) is a neurodevelopmental disorder characterized by inattention or hyperactivity-impulsivity, or both with childhood onset and is associated with deficits across a range of cognitive domains (1, 2). Global prevalence estimates the prevalence of ADHD in adults is approximately 2.5% (3), while children and adolescents have the highest prevalence rate among all age groups, at approximately 5.29% (4). Numerous studies implicated that the development of ADHD is associated with genetic, environmental, psychological factors, or the interaction of these factors (1, 5, 6). In addition to marked cognitive dysfunction, neuroimaging research showed that individuals with ADHD exhibit a mean 2–3-year delay in reaching peak cortical thickness, particularly in the prefrontal cortex, compared to typically developing controls (7, 8). Consequently, patients with ADHD demonstrate marked executive dysfunction, predominantly characterized by deficits in inhibitory control, hyperactivity and impulsivity, and working memory efficiency (2, 9). Aberrant default mode network connectivity further compromises attentional network function, mechanistically contributing to inattention symptomatology (2, 10, 11). Crucially, ADHD significantly impairs social functioning and quality of life among all observed effects (12, 13), establishing the development of effective interventions as a research priority.

Pharmacotherapies such as methylphenidate and amphetamine demonstrate remarkable short-term efficacy in ADHD management (2). However, substantial treatment limitations persist, particularly poor medication tolerability and heightened adherence challenges during adolescence (14). Common adverse effects include appetite suppression, insomnia, dry mouth, and nausea (15). However, alternative treatments, including cognitive training, behavioral therapies, psychological treatments, neurofeedback or dietary interventions, have shown limited efficacy (16–18). Consequently, developing non-pharmacological treatments that effectively enhance neurocognitive performance, ameliorate core ADHD symptoms, and minimize adverse effects represents an imperative research priority.

Non-invasive brain stimulation (NIBS) techniques such as transcranial direct current stimulation (tDCS), transcranial random noise stimulation (tRNS), transcranial alternating current stimulation (tACS), and repetitive transcranial magnetic stimulation (rTMS) are of increasing concern. A recent systematic review and meta-analysis on NIBS for improving cognitive function and clinical symptoms in patients with ADHD has shown that improvement in inhibitory control, working memory, and inattention in tDCS, tACS, and tRNS groups compared with sham groups, while rTMS did not demonstrate significant therapeutic benefits for ADHD symptoms (19). However, another systematic review and meta-analysis observed that tDCS treatment in ADHD has no significant effects on inhibition control and inattention (20). While conventional meta-analyses provide efficacy evidence for NIBS interventions, the relative effectiveness across modalities for ameliorating ADHD-related cognitive deficits and core symptoms remains undetermined. To address this gap, we systematically reviewed randomized controlled trials (RCTs) evaluating NIBS efficacy on cognitive domains and core ADHD symptomatology, subsequently conducting a network meta-analysis. This approach enables direct comparison of five critical outcome domains: inhibitory control, working memory, cognitive flexibility, inattention, hyperactivity and impulsivity. Integrative comparing the efficacy of various NIBS approaches in improving cognitive function and core symptoms in individuals with ADHD, this study aims to provide a theoretical foundation for optimizing and selecting the most effective treatment strategies.

## 2 Methods

This NMA adhered to the Preferred Reporting Items for Systematic Reviews and Meta-Analyses (PRISMA, Supplementary Table S2) guidelines (21). The study protocol was registered in PROSPERO (CRD42025641242) (Supplementary Table S1).

### 2.1 Search strategy

We carried out a systematic search in electronic databases, including PubMed, Embase, Web of Science, the Cochrane Central Register of Controlled Trials (CENTRAL), the China Knowledge Network (CNKI), Wanfang database, and Chongqing Weipu (VIP) from inception to May, 2025. Unpublished registered trials in ClinicalTrials.gov and reference lists of pertinent reviews were also added for a full search. The language and ethnicity of participants in the trials were unfiltered. The complete strategy with search terms adapted for each database is accessible in Supplementary Table S4.

### 2.2 Eligibility criteria

Three independent investigators (LJ, FH, and WXY) selected records according to the screening pipeline filtering through titles, abstracts, and full texts. A fourth reviewer (LXW) was consulted if any discrepancies arose. Studies matching the following criteria were entered in the meta-analysis. (1) Participants: participants with a diagnosis of ADHD; (2) intervention: non-invasive brain stimulation (NIBS) modes, including rTMS, tDCS, and other variants; (3) comparison: sham stimulation or placebo therapy; (4) outcome: standardized tests for assessment of attentional and executive function, such as inhibitory control, working memory, cognitive flexibility, inattention, hyperactivity and impulsivity; (5) study design: clinical randomized controlled trials (RCTs) with human participants.

Studies were excluded if (1) they enrolled non-ADHD subjects; (2) used interventions irrelevant to non-invasive brain stimulation; (3) control groups were not appropriate, e.g., in terms of mismatched age, sex, or severity of disease between groups; (4) the data was incomplete; (5) they were low-quality study types including case series or reports, conference papers, and non-peer-reviewed articles.

### 2.3 Date extraction

Two authors (LJ and FH) independently extracted the following data from the included studies: first author's name, publication year, age (mean), sex (female participant percentage), treatment arms, sample sizes, treatment duration, and stimulation protocol. We contacted the corresponding author if any required data were not reported. A third reviewer (WXY) was consulted if any discrepancies arose. Studies matching the following criteria were entered in the meta-analysis.

### 2.4 Quality assessment

Two authors (LJ and FH) independently assessed the risk of bias in each included trials using the Cochrane risk of bias tool version 2, which consists of the five bias risk domains of the randomization process, deviation from intended intervention, missing outcome data, measurement of the outcome, and selection of reported result. The two investigators sought consensus for disagreements and consulted a third investigator (WXY) when needed.

### 2.5 Outcomes

The primary outcomes included inhibitory control, working memory, cognitive flexibility, inattention, hyperactivity and impulsivity. The inhibitory control was primarily assessed using the Go/No-Go task, flanker task, stop signal task, Stroop task, and inhibiting response (IR) subtest. The working memory was primarily assessed using accuracy in the digit span-backward test. The cognitive flexibility was primarily assessed using perseverative errors in the Wisconsin card sorting test, trail making test, and IR. Inattention was primarily assessed using continuous performance task (CPT), selective attention, the adult ADHD self-report scale (ASRS), visual oddball task and visual attention test, fourth edition (TAVIS- IV) and Swanson, Nolan, and Pelham-IV rating scales (SNAP-IV). And hyperactivity and impulsivity was primarily assessed using ASRS, SNAP-IV, clinician-administered version of the adult ADHD self-report scale (CASRS), CPT and Conner's child behavior scale.

### 2.6 Data synthesis and statistical analysis

We conducted NMA to assess the effects of inhibitory control, working memory, cognitive flexibility, inattention, hyperactivity and impulsivity (continuous variables). We reported continuous variables as standardized mean difference (SMD) due to variations in measurement methods and techniques across different studies, along with their corresponding 95% confidence intervals (CI) and analyses. We used a random-effects model for the analysis, as there is expected heterogeneity among the studies (22). We used STATA (version 17.0) to conduct the NMA.

We utilized the node-splitting method in Stata software to quantify and assess the consistency between indirect comparisons and direct comparisons. If the *P*-value is greater than 0.05, it is considered that the consistency test has passed (23). We also used Stata software to generate and describe the network plot of various interventions. The Surface Under the Cumulative Ranking curve (SUCRA) value used to determine the relative rankings of interventions. SUCRA values range from 0 to 100, with values closer to 0 indicating poorer effectiveness and values closer to 100 indicating greater effectiveness. We checked for potential small study effects and publication bias by conducting comparison-adjusted funnel plots.

## 3 Results

### 3.1 Study selection

**Figure 1:**
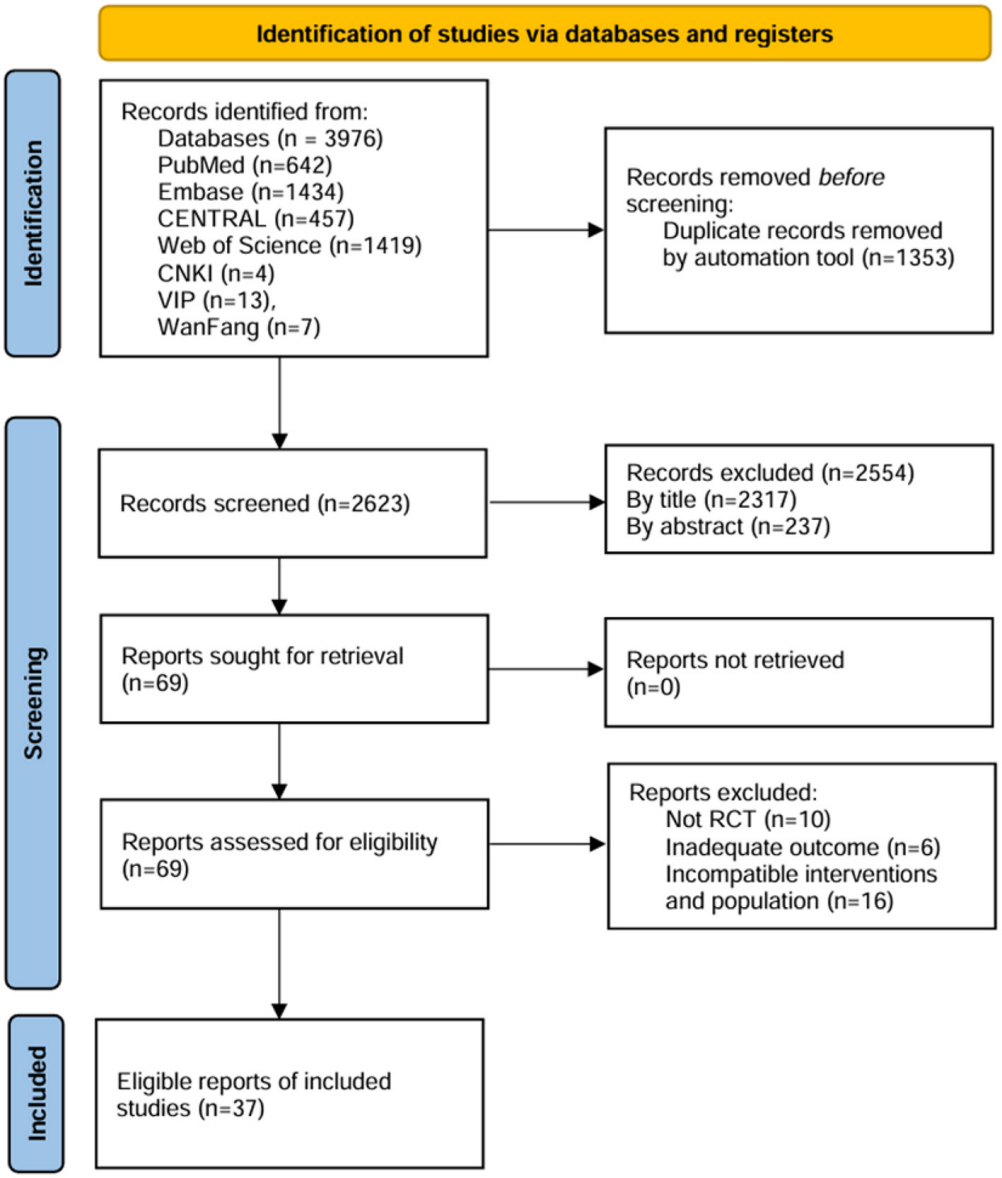
PRISMA flow diagram for study selection.

The study selection process is shown in Figure 1. A total of 3,952 records were identified from the initial search. After eliminating irrelevant records based on titles and abstracts, 69 full-text reports were selected for eligibility assessment. RCTs investigating the application of non-invasive brain stimulation in the context of ADHD are deemed eligible for inclusion. Finally, 37 RCT reports were included in the meta-analysis (24–60).

### 3.2 Study characteristics

The detailed characteristics of individual studies are listed in Supplementary Tables S5–S7. A total of 1,615 patients with ADHD were included in this meta-analysis. There are 33 English studies and four Chinese studies.

Based on the number of trials, tDCS was the most prevailing NIBS for ADHD involving 22 (59.5%) trials, including two studies used HD-tDCS. A total of 10 studies used TMS, including 14 rTMS and one dTMS. Furthermore, there are also three studies on tACS, one on TPS, and one on tRNS. There were 14 articles that studied the effects of NIBS on children with ADHD, four that examined its impact on adolescents, nine that focused on adults with ADHD, and 10 that investigated NIBS across multiple age groups (including children, adolescents, and/or adults). The studies included in this review did not report any other mental illnesses, with only five articles specifically focusing on patients diagnosed with Conduct Disorder (CD) or Oppositional Defiant Disorder (ODD). For the outcome measurements, almost all trials reporting the primary outcomes represented cognitive function and core symptoms in ADHD.

### 3.3 Risk of bias

**Figure 2:**
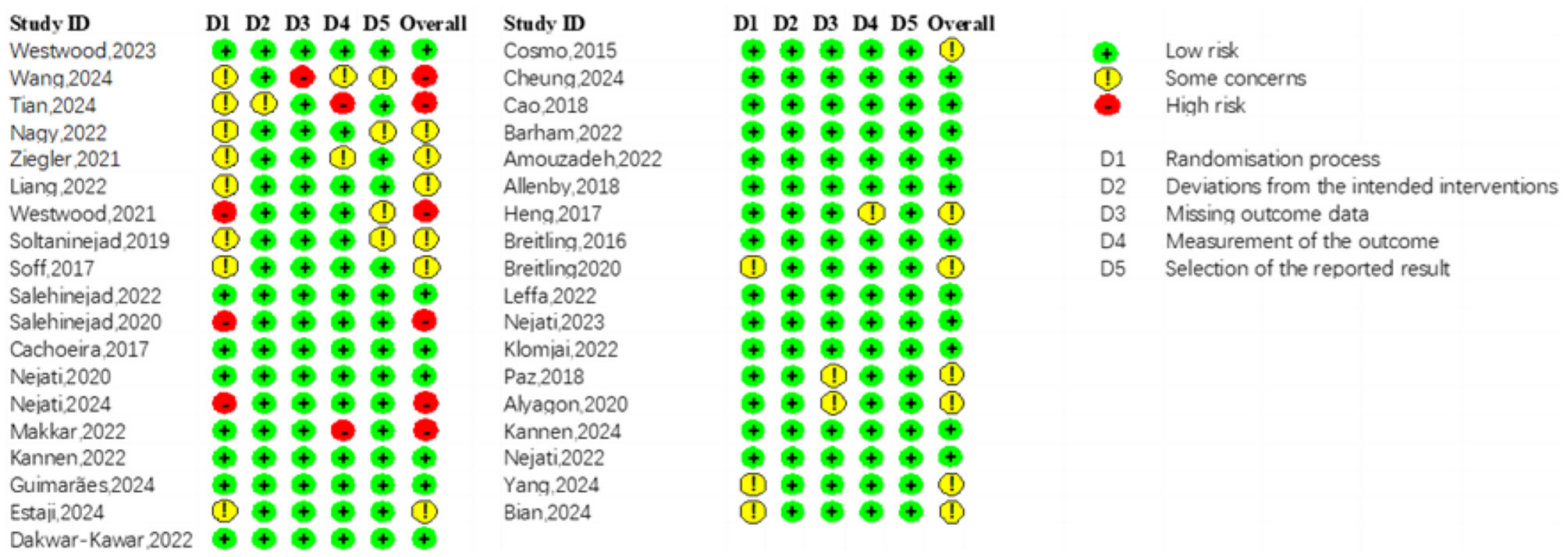
Risk of bias summary of included studies.

The risk of bias of the included studies is summarized in Figure 2. Across the five domains of the PRISMA RoB2 tool, the randomization process was the domain with the highest risk of bias. Measurement of the outcome were also ranked high in a small proportion of studies. Overall, the risk of bias in most included trials was rated as some concerns. The calculated Cohen's κ for authors' consensus on the risk of bias was 0.76(κ > 0.6), indicating substantial inter-rater agreement.

### 3.4 Network geometry of interventions

Overall, the participant enrollment figures for the five primary outcomes are as follows, Inhibitory Control (*n* = 520), Working Memory (*n* = 243), Cognitive Flexibility (*n* = 244), Inattention (*n* = 699), Hypersensitivity and Impulsivity (*n* = 274). Fourteen tDCS protocols, including (1) anodal tDCS over the left DLPFC plus cathodal tDCS over the right DLPFC (atDCS_F3 + ctDCS_F4), (2) anodal tDCS over the left DLPFC plus cathodal tDCS over the right supraorbital area 1.5 mA (atDCS_FP2 + ctDCS_F3^*^1.5), (3) high-definition anodal transcranial direct current stimulation over the vertex 0.5 mA (HD_atDCS_Cz^*^0.5), (4) high-definition anodal transcranial direct current stimulation over the vertex 0.25 mA (HD_atDCS_Cz^*^0.25), (5) anodal tDCS over the right DLPFC (atDCS_F8), (6) cathodal tDCS over the posterior to the left mastoid (ctDCS_P7), (7) anodal tDCS over the left DLPFC plus cathodal tDCS over the right DLPFC 2 mA (atDCS_F3 + ctDCS_F4^*^2), (8) anodal tDCS over the left DLPFC plus cathodal tDCS over the left supraorbital area (atDCS_F3 + ctDCS_FP1), (9) anodal tDCS over the right inferior frontal cortex (rIFC) plus cathodal tDCS over the right supraorbital area (atDCS_F8 + ctDCS_FP1), (10) anodal tDCS over the left DLPFC plus cathodal tDCS over the right supraorbital area (atDCS_F3 + ctDCS_FP2), (11) cathodal tDCS over the left DLPFC plus anodal tDCS over the right supraorbital area (ctDCS_F3 + atDCS_FP2), (12) anodal tDCS over the right DLPFC plus cathodal tDCS over the left DLPFC 2 mA (atDCS_F4 + ctDCS_F3^*^2), (13) anode over the left DLPFC F3 and cathode over the vertext Cz (atDCS_F3 + ctDCS_Cz), and (14) anodal tDCS over the left DLPFC plus cathodal tDCS over the left supraorbital area 2 mA (atDCS_F3 + ctDCS_FP1^*^2); one TPS protocols, including TPS; one tRNS procotols, including tRNS; three tACS protocols, including (1) tACS, (2) tACS^*^8; and (3) tACS^*^10; two rTMS protocols, including HF_rTMS_rDLPEC^*^10, and rTMS_M1. Table 1 demonstrates the explanation of interventions.

**Table 1:** Explanation of interventions.

**tDCS (*N* = 14)**	**a** = **anodal; c** = cathodal; tDCS is Stimulation Modality; The part after the “_” symbol indicates the electrode placements; The value after “^*^” indicates stimulus intensity (mA); if unspecified, it defaults to 1mA		
**No**.	**Abbreviation**	**Full term**	**The electrode placements**
1	atDCS_F3 + ctDCS_F4	anodal tDCS over the left DLPFC plus cathodal tDCS over the right DLPFC	F3 = left frontal 3; F4 = right frontal 4
2	atDCS_FP2 + ctDCS_F3^*^1.5	anodal tDCS over the left DLPFC plus cathodal tDCS over the right supraorbital area 1.5mA	FP2 = right frontopolar 2; F3 = left frontal 3
3	HD_atDCS_Cz^*^0.5	High-definition anodal transcranial direct current stimulation over the vertex 0.5mA	HD = high-definition; Cz = central region
4	HD_atDCS_Cz^*^0.25	High-definition anodal transcranial direct current stimulation over the vertex 0.25mA	HD = high-definition; Cz = central region
5	atDCS_F8	anodal tDCS over the right DLPFC	F8 = right frontotemporal 8
6	ctDCS_P7	cathodal tDCS over the posterior to the left mastoid	P7 = left parietal 7
7	atDCS_F3 + ctDCS_F4^*^2	anodal tDCS over the left DLPFC plus cathodal tDCS over the right DLPFC 2mA	F3 = left frontal 3; F4 = right frontal 4
8	atDCS_F3 + ctDCS_FP1	anodal tDCS over the left DLPFC plus cathodal tDCS over the left supraorbital area	F3 = left frontal 3; FP1 = left frontopolar 1
9	atDCS_F8 + ctDCS_FP1	anodal tDCS over the rIFC plus cathodal tDCS over the right supraorbital area	F8 = right frontotemporal 8; FP1 = left frontopolar 1
10	atDCS_F3 + ctDCS_FP2	anodal tDCS over the left DLPFC plus cathodal tDCS over the right supraorbital area	F3 = left frontal 3; FP2 = right frontopolar 2
11	ctDCS_F3 + atDCS_FP2	cathodal tDCS over the left DLPFC plus anodal tDCS over the right supraorbital area	F3 = left frontal 3; FP2 = right frontopolar 2
12	atDCS_F4 + ctDCS_F3^*^2	anodal tDCS over the right DLPFC plus cathodal tDCS over the left DLPFC 2mA	F4 = right frontal 4; F3 = left frontal 3
13	atDCS_F3 + ctDCS_Cz	anode over the left DLPFC F3 and cathode over the vertext Cz	F3 = left frontal 3; Cz = central region
14	atDCS_F3 + ctDCS_FP1^*^2	anodal tDCS over the left DLPFC plus cathodal tDCS over the left supraorbital area 2mA	F3 = left frontal 3; FP1 = left frontopolar 1
**TPS**	**TPS is stimulation modality**		
**No**.	**Abbreviation**	**Full term**	
1	TPS	Transcranial Pulse Stimulation	
**tRNS**	**tRNS is stimulation modality**		
**No**.	**Abbreviation**	**Full term**	
1	tRNS	Transcranial Random Noise Stimulation	
**tACS**	**tACS is Stimulation Modality; The value after “** ^*^ **” indicates stimulus intensity**		
**No**.	**Abbreviation**	**Full term**	
1	tACS	Transcranial Alternating Current Stimulation	
2	tACS^*^8	Transcranial Alternating Current Stimulation 8 Hz	
3	tACS^*^10	Transcranial Alternating Current Stimulation 10 Hz	
**rTMS**	**rTMS is Stimulation Modality; The part after the “_” symbol indicates the electrode placements; The value after “** ^*^ **” indicates stimulus intensity**		
**No**.	**Abbreviation**	**Full term**	**The electrode placements**
1	HF_rTMS_rDLPEC^*^10	rTMS over the right dorsolateral prefrontal cortex 10Hz	HF = high-frequency; rTMS = repetitive Transcranial Magnetic Stimulation; rDLPEC=the right dorsolateral prefrontal cortex
2	rTMS_M1	rTMS over the left thumb motor cortex area	rTMS=repetitive Transcranial Magnetic Stimulation; M1=the left thumb motor cortex area

**Figure 3:**
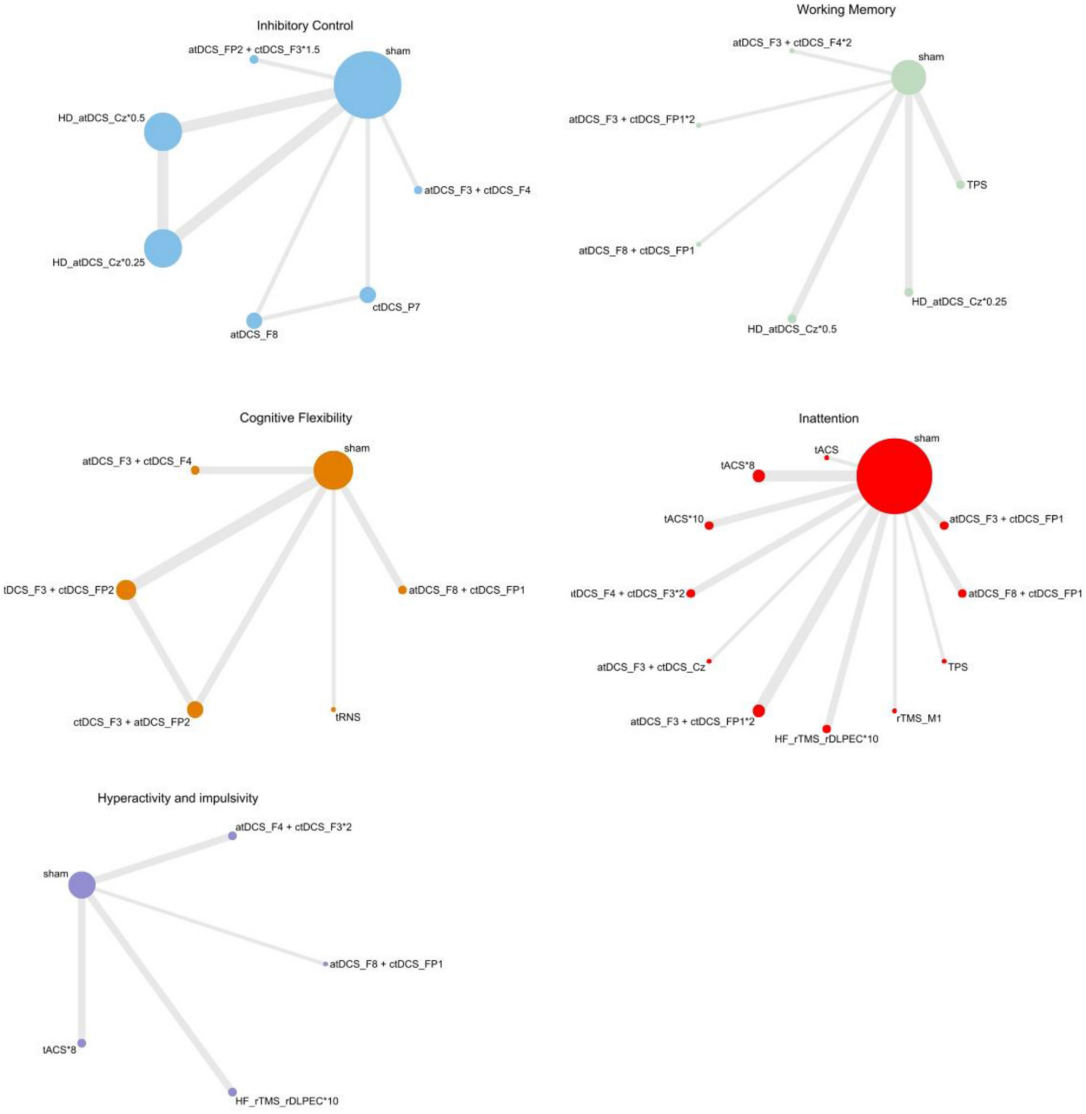
Network results of individual symptoms. a, anodal; c, cathodal; Cz, central region; F3, left frontal 3; F4, right frontal 4; F8, right frontotemporal 8; FP1, left frontopolar 1; FP2, right frontopolar 2; HD, high-definition; HF, high-frequency; M1, primary motor cortex; P7, left parietal 7; rDLPEC, right dorsolateral prefrontal cortex; rTMS, repetitive transcranial magnetic stimulation; tACS, transcranial alternating current stimulation; tDCS, transcranial direct current stimulation; TPS, transcranial pulse stimulation; tRNS, transcranial random noise stimulation.

Refined networks subdivided by targeted brain regions are presented in Figure 3, including seven interventions for Inhibitory Control (eight comparisons), Working Memory (seven interventions, six comparisons), Cognitive Flexibility (six interventions, six comparisons), Inattention (12 interventions, 11 comparisons), Hypersensitivity and Impulsivity (five interventions, four comparisons).

### 3.5 Efficacy

#### 3.5.1 Inhibitory control

**Figure 4:**
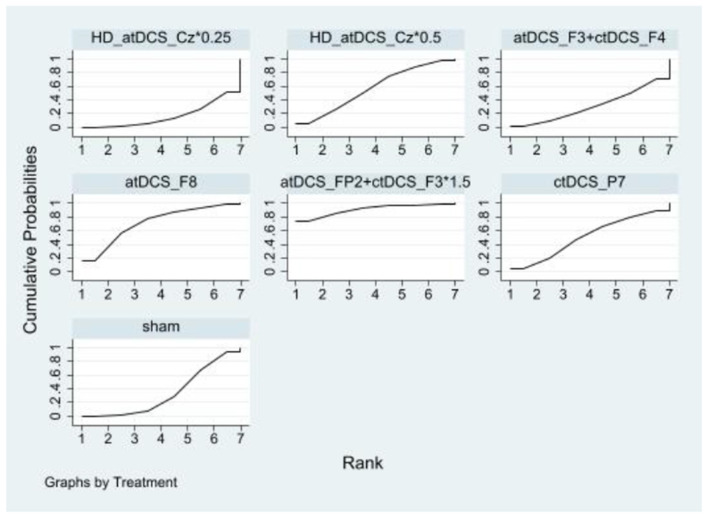
Ranking results of inhibitory control.

In inhibitory control, there were no statistically significant differences between the NIBS intervention and sham controls regarding improvements. However, a significant difference was observed between the HD_atDCS_Cz^*^0.25 and atDCS_FP2+ctDCS_F3^*^1.5 conditions. The effects sizes of improving inhibitory control for the top four NIBS interventions were atDCS_FP2+ctDCS_F3^*^1.5 [SMD = −0.87, 95% CI: (−1.80, 0.07)], atDCS_F8 [SMD = −0.40, 95% CI: (−1.05, 0.25)], HD_atDCS_Cz^*^0.5 [SMD = −0.20, 95% CI: (−0.69, 0.29)] and ctDCS_P7 [SMD = −0.17, 95% CI: (−0.82, 0.48)]. According to SUCRA analysis, the top four interventions for improving inhibitory control were atDCS_FP2+ctDCS_F3^*^1.5 (90.8%), atDCS_F8 (71.8%), HD_atDCS_Cz^*^0.5 (56.5%), and ctDCS_P7 (50.6%; Figure 4 and Table 2).

**Table 2:** Ranking results of inhibitory control.

atDCS_FP2+ctDCS_F3^*^1.5	**atDCS_F8**	HD_atDCS_Cz^*^0.5	**ctDCS_P7**	**sham**	**atDCS_F3+ctDCS_F4**	HD_atDCS_Cz^*^0.25
atDCS_FP2+ctDCS_F3^*^1.5	0.47 (−0.67, 1.60)	0.67 (−0.38, 1.72)	0.70 (−0.44, 1.83)	0.87 (−0.07, 1.80)	0.92 (−0.19, 2.03)	1.04 (−0.00, 2.09)
−0.47 (−1.60, 0.67)	atDCS_F8	0.20 (−0.61, 1.02)	0.23 (−0.41, 0.88)	0.40 (−0.25, 1.05)	0.45 (−0.43, 1.34)	0.58 (−0.22, 1.38)
−0.67 (−1.72, 0.38)	−0.20 (−1.02, 0.61)	HD_atDCS_Cz^*^0.5	0.03 (−0.78, 0.84)	0.20 (−0.29, 0.69)	0.25 (−0.53, 1.03)	0.37 (−0.13, 0.88)
−0.70 (−1.83, 0.44)	−0.23 (−0.88, 0.41)	−0.03 (−0.84, 0.78)	ctDCS_P7	0.17 (−0.48, 0.82)	0.22 (−0.66, 1.10)	0.35 (−0.46, 1.15)
−0.87 (−1.80, 0.07)	−0.40 (−1.05, 0.25)	−0.20 (−0.69, 0.29)	−0.17 (−0.82, 0.48)	sham	0.05 (−0.55, 0.65)	0.18 (−0.30, 0.65)
−0.92 (−2.03, 0.19)	−0.45 (−1.34, 0.43)	−0.25 (−1.03, 0.53)	−0.22 (−1.10, 0.66)	−0.05 (−0.65, 0.55)	atDCS_F3+ctDCS_F4	0.12 (−0.64, 0.89)
−1.04 (−2.09, 0.00)	−0.58 (−1.38, 0.22)	−0.37 (−0.88, 0.13)	−0.35 (−1.15, 0.46)	−0.18 (−0.65, 0.30)	−0.12 (−0.89, 0.64)	HD_atDCS_Cz^*^0.25

#### 3.5.2 Working memory

**Figure 5:**
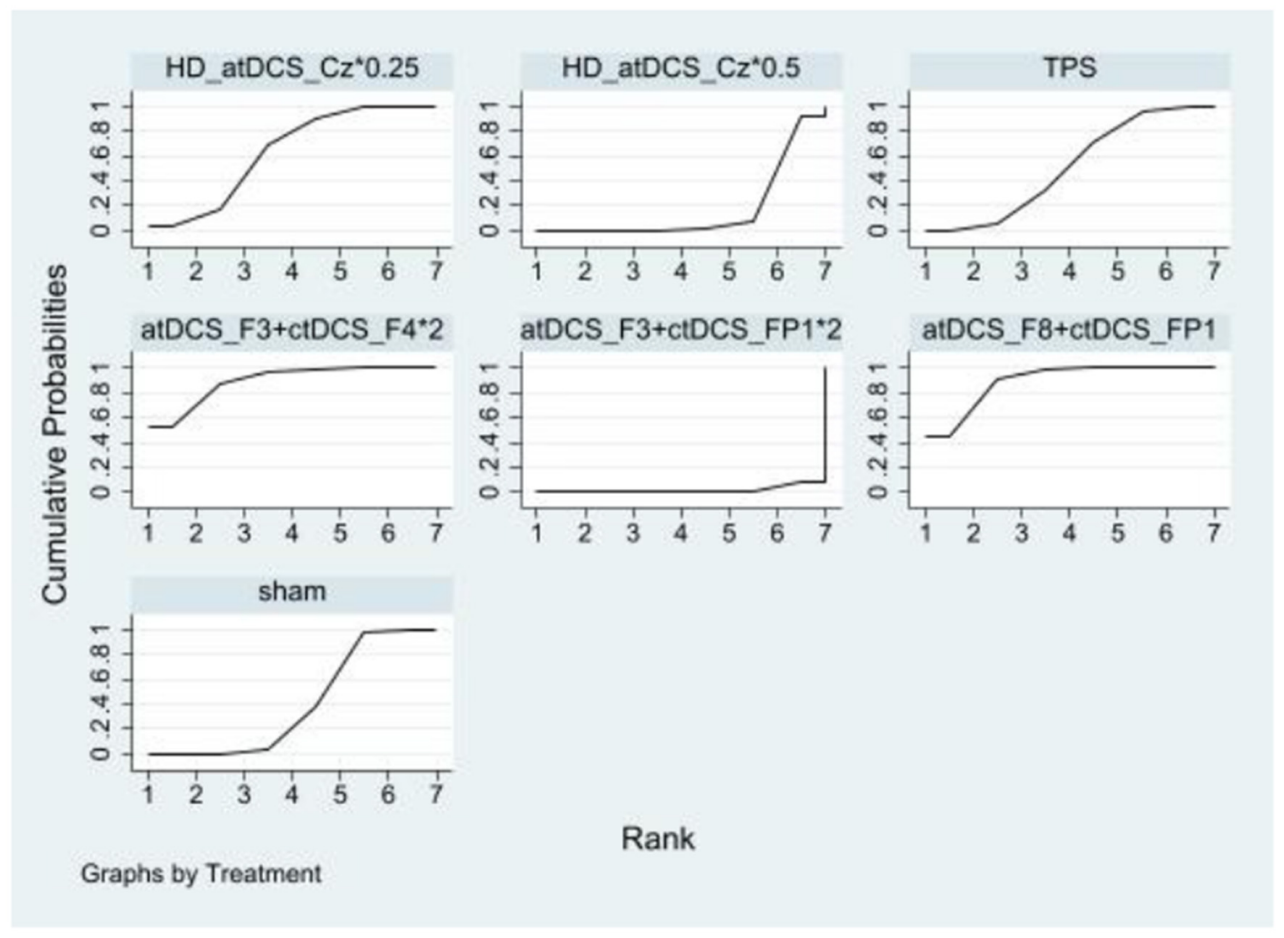
Ranking results of working memory.

Compared to the sham controls, atDCS_F3+ctDCS_F4^*^2 [SMD = 0.95, 95% CI: (0.05, 1.84)] and atDCS_F8+ctDCS_FP1 [SMD = 0.86, 95% CI: (0.28, 1.45)] significantly improved working memory. The effects sizes of improving working memory for the top four NIBS interventions were atDCS_F3+ctDCS_F4^*^2, atDCS_F8+ctDCS_FP1, HD_atDCS_Cz^*^0.25 [SMD = 0.32, 95% CI: (−0.25, 0.89)] and TPS [SMD = 0.14, 95% CI: (−0.35, 0.63)]. According to SUCRA analysis, the top four interventions for improving working memory were atDCS_F3+ctDCS_F4^*^2 (89.3%), atDCS_F8+ctDCS_FP1 (88.6%), HD_atDCS_Cz^*^0.25 (62.5%) and TPS (51.1%; Figure 5 and Table 3).

**Table 3:** Ranking results of working memory.

atDCS_F3+ctDCS_F4^*^2	**atDCS_F8+ctDCS_FP1**	HD_atDCS_Cz^*^0.25	**TPS**	**sham**	HD_atDCS_Cz^*^0.5	atDCS_F3+ctDCS_FP1^*^2
atDCS_F3+ctDCS_F4^*^2	−0.08 (−1.15, 0.99)	−0.62 (−1.69, 0.44)	−0.81 (−1.83, 0.21)	−0.95 (−1.84, −0.05)	−1.52 (−2.60, −0.43)	−2.47 (−3.98, −0.97)
0.08 (−0.99, 1.15)	atDCS_F8+ctDCS_FP1	−0.54 (−1.36, 0.27)	−0.73 (−1.49, 0.04)	−0.86 (−1.45, −0.28)	−1.44 (−2.29, −0.59)	−2.39 (−3.74, −1.05)
0.62 (−0.44, 1.69)	0.54 (−0.27, 1.36)	HD_atDCS_Cz^*^0.25	−0.18 (−0.94, 0.57)	−0.32 (−0.89, 0.25)	−0.89 (−1.74, −0.05)	−1.85 (−3.19, −0.51)
0.81 (−0.21, 1.83)	0.73 (−0.04, 1.49)	0.18 (−0.57, 0.94)	TPS	−0.14 (−0.63, 0.35)	−0.71 (−1.50, 0.08)	−1.67 (−2.97, −0.36)
0.95 (0.05, 1.84)	0.86 (0.28, 1.45)	0.32 (−0.25, 0.89)	0.14 (−0.35, 0.63)	Sham	−0.57 (−1.19, 0.04)	−1.53 (−2.74, −0.32)
1.52 (0.43, 2.60)	1.44 (0.59, 2.29)	0.89 (0.05, 1.74)	0.71 (−0.08, 1.50)	0.57 (−0.04, 1.19)	HD_atDCS_Cz^*^0.5	−0.95 (−2.31, 0.40)
2.47 (0.97, 3.98)	2.39 (1.05, 3.74)	1.85 (0.51, 3.19)	1.67 (0.36, 2.97)	1.53 (0.32, 2.74)	0.95 (−0.40, 2.31)	atDCS_F3+ctDCS_FP1^*^2

#### 3.5.3 Cognitive flexibility

**Figure 6:**
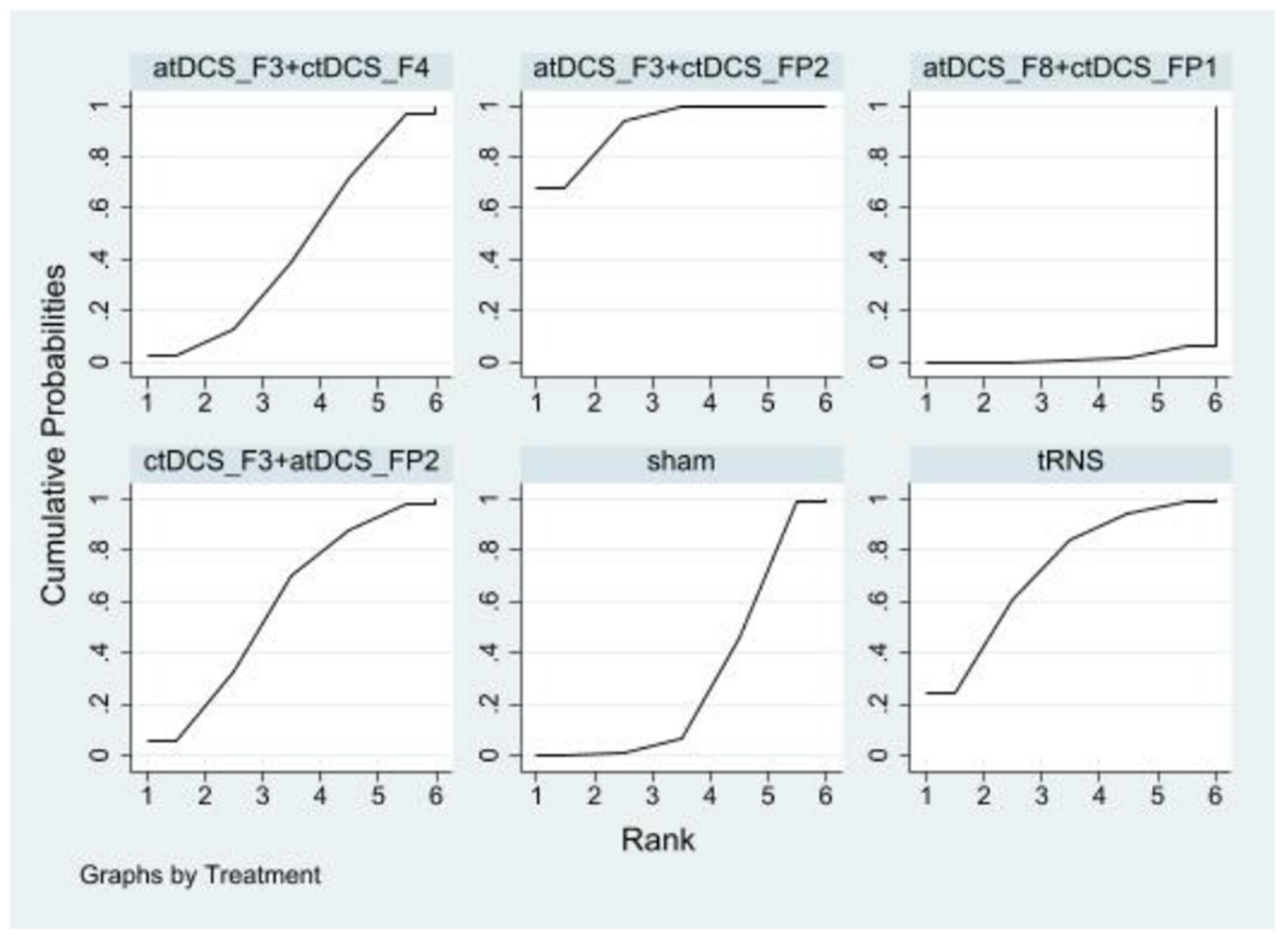
Ranking results of cognitive flexibility.

In cognitive flexibility, only atDCS_F3+ctDCS_FP2 [SMD = −0.76, 95% CI: (−1.31, −0.21)] showed a statistically significant improvement compared to the sham control group. The effect sizes of improving cognitive flexibility for the top four NIBS interventions were atDCS_F3+ctDCS_FP2, tRNS [SMD = −0.49. 95% CI: (−1.15, 0.18)], ctDCS_F3+atDCS_FP2 [SMD = −0.32, 95% CI: (−0.92, 0.28)], and atDCS_F3+ctDCS_F4 [SMD = −0.13, 95% CI: (−0.64, 0.38)]. According to SUCRA analysis, the top four interventions for improving cognitive flexibility were atDCS_F3+ctDCS_FP2 (92.2%), tRNS (72.5%), ctDCS_F3+atDCS_FP2 (58.7%), and atDCS_F3+ctDCS_F4 (44.6%; Figure 6 and Table 4).

**Table 4:** Ranking results of cognitive flexibility.

**atDCS_F3+ ctDCS_FP2**	**tRNS**	**ctDCS_F3+ atDCS_FP2**	**atDCS_F3+ ctDCS_F4**	**sham**	**atDCS_F8+ ctDCS_FP1**
atDCS_F3+ctDCS_FP2	0.27 (−0.59, 1.13)	0.44 (−0.17, 1.04)	0.63 (−0.12, 1.37)	0.76 (0.21, 1.31)	1.26 (0.54, 1.98)
−0.27 (−1.13, 0.59)	tRNS	0.17 (−0.73, 1.07)	0.36 (−0.48, 1.19)	0.49 (−0.18, 1.15)	0.99 (0.18, 1.80)
−0.44 (−1.04, 0.17)	−0.17 (−1.07, 0.73)	ctDCS_F3+atDCS_FP2	0.19 (−0.60, 0.98)	0.32 (−0.28, 0.92)	0.82 (0.06, 1.59)
−0.63 (−1.37, 0.12)	−0.36 (−1.19, 0.48)	−0.19 (−0.98, 0.60)	atDCS_F3+ctDCS_F4	0.13 (−0.38, 0.64)	0.63 (−0.06, 1.32)
−0.76 (−1.31, −0.21)	−0.49 (−1.15, 0.18)	−0.32 (−0.92, 0.28)	−0.13 (−0.64, 0.38)	sham	0.50 (0.03, 0.97)
−1.26 (−1.98, −0.54)	−0.99 (−1.80, −0.18)	−0.82 (−1.59, −0.06)	−0.63 (−1.32, 0.06)	−0.50 (−0.97, −0.03)	atDCS_F8+ctDCS_FP1

#### 3.5.4 Inattention

**Figure 7:**
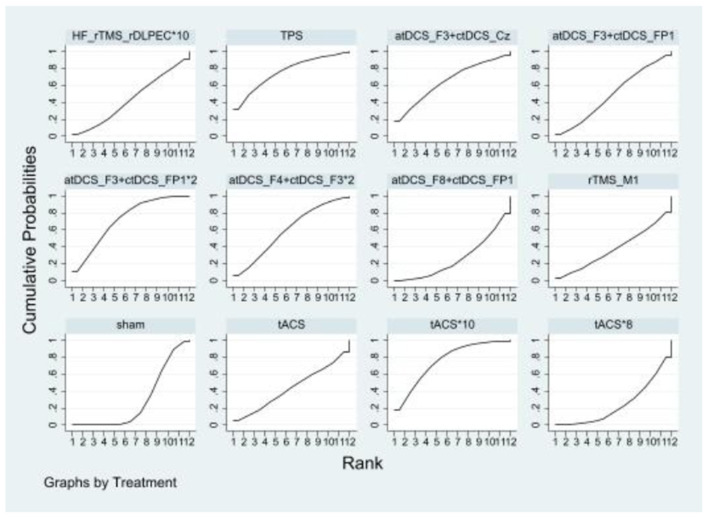
Ranking results of inattention.

Compared to the sham controls, none of the NIBS interventions significantly improved inattention. Only the comparison between TPS and tACS^*^10 is close to being significant when compared to sham. The effects sizes of improving inattention for the top nine NIBS interventions were TPS [SMD = −2.62, 95% CI: (−6.35, 1.12)], tACS^*^10 [SMD = −2.35, 95% CI: (−5.00, 0.30)], atDCS_F3+ctDCS_FP1^*^2 [SMD = −2.09, 95% CI: (−4.32, 0.15)], atDCS_F3+ctDCS_Cz [SMD = −1.88, 95% CI: (−5.59, 1.83)], atDCS_F4+ctDCS_F3^*^2 [SMD = −1.37, 95% CI: (−4.02, 1.28)], atDCS_F3+ctDCS_FP1 [SMD = −0.91, 95% CI: (−3.48, 1.67)], tACS [SMD = −0.60, 95% CI: (−4.28, 3.07)], HF_rTMS_rDLPEC^*^10 [SMD = −0.61, 95% CI: (−3.35, 2.13)] and rTMS_M1 [SMD = −0.25, 95% CI: (−3.91, 3.41)]. According to SUCRA analysis, the top nine interventions for improving inattention were TPS (76.0%), tACS^*^10 (75.5%), atDCS_F3+ctDCS_FP1^*^2 (71.6%), atDCS_F3+ctDCS_Cz (64.9%), atDCS_F4+ctDCS_F3^*^2 (59.4%), atDCS_F3+ctDCS_FP1 (49.4%), tACS (43.4%), HF_rTMS_rDLPEC^*^10 (43.7%), rTMS_M1 (37.5%; Figure 7 and Table 5).

**Table 5:** Ranking results of inattention.

**TPS**	tACS^*^10	atDCS_F3+ ctDCS_FP1^*^2	**atDCS_F3+ ctDCS_Cz**	atDCS_F4+ ctDCS_F3^*^2	**atDCS_F3+ ctDCS_FP1**	**tACS**	HF_rTMS_ rDLPEC^*^10	**rTMS_M1**	**sham**	**atDCS_F8+ ctDCS_FP1**	tACS^*^8
TPS	0.27 (−4.31, 4.85)	0.53 (−3.82, 4.88)	0.74 (−4.53, 6.00)	1.25 (−3.33, 5.83)	1.71 (−2.83, 6.25)	2.01 (−3.23, 7.26)	2.01 (−2.62, 6.64)	2.37 (−2.86, 7.60)	2.62 (−1.12, 6.35)	2.85 (−1.66, 7.36)	2.96 (−1.34, 7.26)
−0.27 (−4.85, 4.31)	tACS^*^10	0.26 (−3.20, 3.73)	0.47 (−4.09, 5.03)	0.98 (−2.76, 4.73)	1.44 (−2.25, 5.14)	1.75 (−2.79, 6.28)	1.74 (−2.07, 5.55)	2.10 (−2.42, 6.62)	2.35 (−0.30, 5.00)	2.58 (−1.08, 6.25)	2.69 (−0.71, 6.09)
−0.53 (−4.88, 3.82)	−0.26 (−3.73, 3.20)	atDCS_F3+ ctDCS_FP1^*^2	0.21 (−4.12, 4.54)	0.72 (−2.75, 4.18)	1.18 (−2.23, 4.59)	1.48 (−2.82, 5.78)	1.48 (−2.06, 5.01)	1.84 (−2.45, 6.13)	2.09 (−0.15, 4.32)	2.32 (−1.06, 5.70)	2.43 (−0.66, 5.51)
−0.74 (−6.00, 4.53)	−0.47 (−5.03, 4.09)	−0.21 (−4.54, 4.12)	atDCS_F3+ ctDCS_Cz	0.51 (−4.05, 5.07)	0.97 (−3.54, 5.49)	1.28 (−3.95, 6.50)	1.27 (−3.34, 5.88)	1.63 (−3.58, 6.84)	1.88 (−1.83, 5.59)	2.11 (−2.38, 6.61)	2.22 (−2.06, 6.50)
−1.25 (−5.83, 3.33)	−0.98 (−4.73, 2.76)	−0.72 (−4.18, 2.75)	−0.51 (−5.07, 4.05)	atDCS_F4+ ctDCS_F3^*^2	0.46 (−3.23, 4.16)	0.77 (−3.76, 5.30)	0.76 (−3.05, 4.57)	1.12 (−3.40, 5.64)	1.37 (−1.28, 4.02)	1.60 (−2.06, 5.27)	1.71 (−1.69, 5.11)
−1.71 (−6.25, 2.83)	−1.44 (−5.14, 2.25)	−1.18 (−4.59, 2.23)	−0.97 (−5.49, 3.54)	−0.46 (−4.16, 3.23)	atDCS_F3+ ctDCS_FP1	0.30 (−4.19, 4.79)	0.30 (−3.46, 4.06)	0.66 (−3.82, 5.14)	0.91 (−1.67, 3.48)	1.14 (−2.47, 4.76)	1.25 (−2.10, 4.59)
−2.01 (−7.26, 3.23)	−1.75 (−6.28, 2.79)	−1.48 (−5.78, 2.82)	−1.28 (−6.50, 3.95)	−0.77 (−5.30, 3.76)	−0.30 (−4.79, 4.19)	tACS	−0.01 (−4.59, 4.58)	0.35 (−4.84, 5.54)	0.60 (−3.07, 4.28)	0.84 (−3.63, 5.30)	0.94 (−3.31, 5.19)
−2.01 (−6.64, 2.62)	−1.74 (−5.55, 2.07)	−1.48 (−5.01, 2.06)	−1.27 (−5.88, 3.34)	−0.76 (−4.57, 3.05)	−0.30 (−4.06, 3.46)	0.01 (−4.58, 4.59)	HF_rTMS_ rDLPEC^*^10	0.36 (−4.22, 4.94)	0.61 (−2.13, 3.35)	0.84 (−2.89, 4.58)	0.95 (−2.53, 4.42)
−2.37 (−7.60, 2.86)	−2.10 (−6.62, 2.42)	−1.84 (−6.13, 2.45)	−1.63 (−6.84, 3.58)	−1.12 (−5.64, 3.40)	−0.66 (−5.14, 3.82)	−0.35 (−5.54, 4.84)	−0.36 (−4.94, 4.22)	rTMS_M1	0.25 (−3.41, 3.91)	0.48 (−3.97, 4.94)	0.59 (−3.65, 4.83)
−2.62 (−6.35, 1.12)	−2.35 (−5.00, 0.30)	−2.09 (−4.32, 0.15)	−1.88 (−5.59, 1.83)	−1.37 (−4.02, 1.28)	−0.91 (−3.48, 1.67)	−0.60 (−4.28, 3.07)	−0.61 (−3.35, 2.13)	−0.25 (−3.91, 3.41)	sham	0.23 (−2.31, 2.77)	0.34 (−1.79, 2.47)
−2.85 (−7.36, 1.66)	−2.58 (−6.25, 1.08)	−2.32 (−5.70, 1.06)	−2.11 (−6.61, 2.38)	−1.60 (−5.27, 2.06)	−1.14 (−4.76, 2.47)	−0.84 (−5.30, 3.63)	−0.84 (−4.58, 2.89)	−0.48 (−4.94, 3.97)	−0.23 (−2.77, 2.31)	atDCS_F8+ ctDCS_FP1	0.11 (−3.21, 3.42)
−2.96 (−7.26, 1.34)	−2.69 (−6.09, 0.71)	−2.43 (−5.51, 0.66)	−2.22 (−6.50, 2.06)	−1.71 (−5.11, 1.69)	−1.25 (−4.59, 2.10)	−0.94 (−5.19, 3.31)	−0.95 (−4.42, 2.53)	−0.59 (−4.83, 3.65)	−0.34 (−2.47, 1.79)	−0.11 (−3.42, 3.21)	tACS^*^8

#### 3.5.5 Hyperactivity and impulsivity

**Figure 8:**
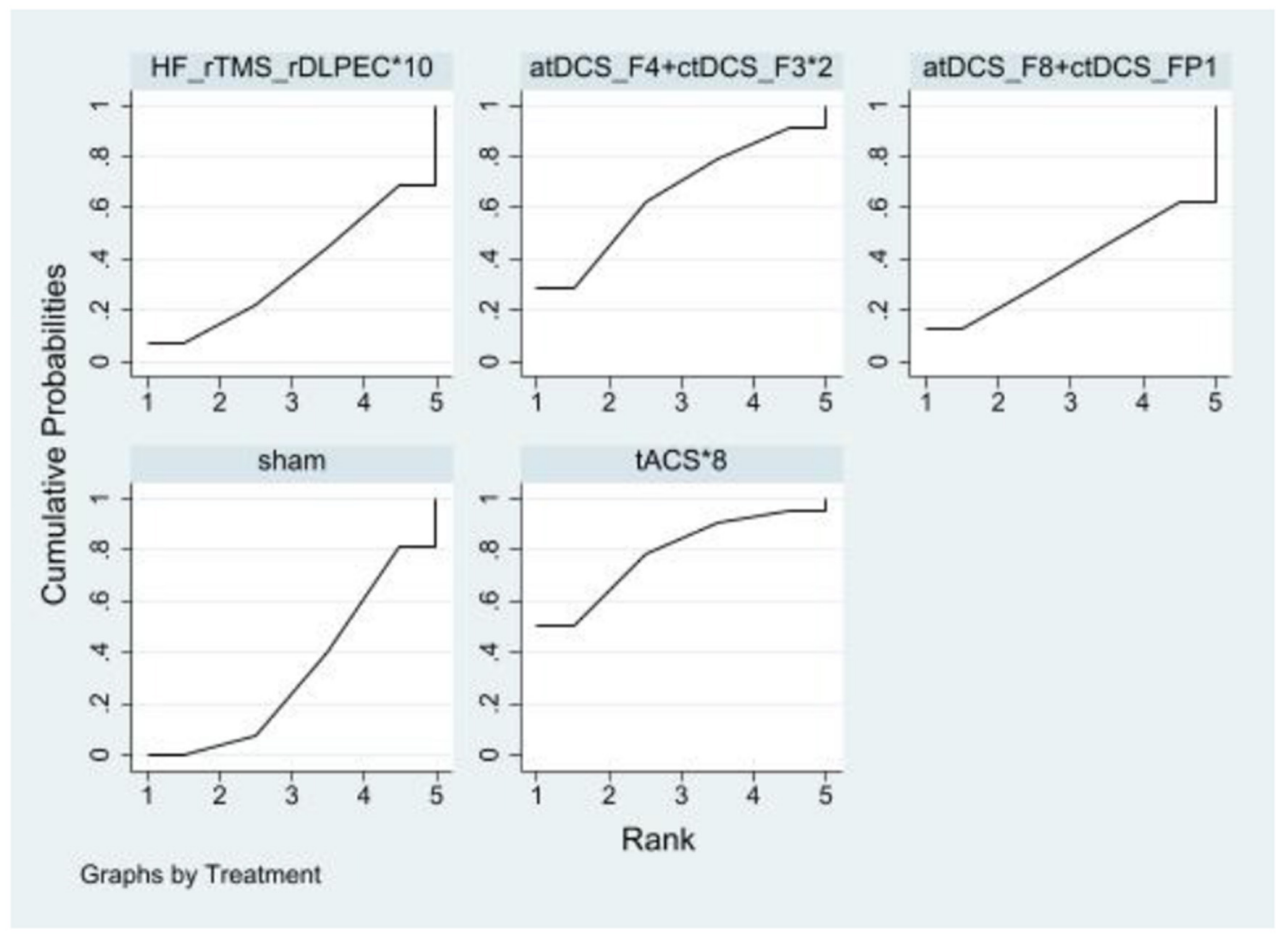
Ranking results of hyperactivity and impulsivity.

Compared to the sham controls, none of the NIBS interventions significantly improved hyperactivity and impulsivity. The effects sizes of improving hyperactivity and impulsivity for the top four NIBS interventions were tACS^*^8 [SMD = −0.66, 95% CI: (−1.60, 0.28)], atDCS_F4+ctDCS_F3^*^2 [SMD = −0.42, 95% CI: (−1.38, 0.54)], atDCS_F8+ctDCS_FP1 [SMD = −0.00, 95% CI: (−1.23, 1.22)] and HF_rTMS_rDLPEC^*^10 [SMD = −0.01, 95% CI: (−0.88, 0.87)]. According to SUCRA analysis, the top four interventions for improving hyperactivity and impulsivity were tACS^*^8 (78.8%), atDCS_F4+ctDCS_F3^*^2 (65.5%), atDCS_F8+ctDCS_FP1 (37.5%), HF_rTMS_rDLPEC^*^10 (35.7%; Figure 8 and Table 6).

**Table 6:** Ranking results of hyperactivity and impulsivity.

tACS^*^8	atDCS_F4+ctDCS_F3^*^2	**atDCS_F8+ctDCS_FP1**	HF_rTMS_rDLPEC^*^10	**sham**
tACS^*^8	0.24 (−1.10, 1.58)	0.66 (−0.89, 2.20)	0.66 (−0.63, 1.94)	0.66 (−0.28, 1.60)
−0.24 (−1.58, 1.10)	atDCS_F4+ctDCS_F3^*^2	0.42 (−1.14, 1.98)	0.42 (−0.88, 1.72)	0.42 (−0.54, 1.38)
−0.66 (−2.20, 0.89)	−0.42 (−1.98, 1.14)	atDCS_F8+ctDCS_FP1	−0.00 (−1.51, 1.50)	0.00 (−1.22, 1.23)
−0.66 (−1.94, 0.63)	−0.42 (−1.72, 0.88)	0.00 (−1.50, 1.51)	HF_rTMS_rDLPEC^*^10	0.01 (−0.87, 0.88)
−0.66 (−1.60, 0.28)	−0.42 (−1.38, 0.54)	−0.00 (−1.23, 1.22)	−0.01 (−0.88, 0.87)	sham

## 4 Discussion

This network meta-analysis (NMA) provides a comprehensive evaluation of various non-invasive brain stimulation (NIBS) modalities for reducing ADHD symptoms. Based on 37 RCTs, the results showed that atDCS_F3 + ctDCS_FP2 improved cognitive flexibility compared to sham controls. For working memory, atDCS_F3 + ctDCS_F4 demonstrated statistically significant improvement relative to sham stimulations. No NIBS interventions significantly improved inhibitory control or hypersensitivity/impulsivity compared to sham stimulation. Although previous meta-analyses have demonstrated the efficacy of individual NIBS methods for treating ADHD, this is the first NMA to comprehensively compare the effectiveness of different NIBS modalities on inhibitory control, working memory, cognitive flexibility, inattention, and hyperactivity/impulsivity.

### 4.1 Domain specific rankings across cognition aspects

We focus on examining the effectiveness of various NIBS treatments in improving cognitive function in ADHD. Therefore, we rank the efficacy of these functions in terms of ADHD. A review indicated that transcranial direct current stimulation (tDCS) is effective in enhancing cognitive function, particularly highlighting the significant benefits of anodal transcranial direct current stimulation (a-tDCS) on working memory and cognitive flexibility (61). In addition, tDCS has been used to enhance cognitive, emotional, and social functioning in both healthy individuals and Alzheimer's disease (62). The advantageous mechanism of tDCS, a weak direct electrical current is delivered through two electrodes placed on the scalp (one anode, one cathode), generating subthreshold, polarity-dependent shifts in resting membrane potentials in underlying brain regions (63). The net increase (predominantly under the anode) or decrease (predominantly under the cathode) in neuronal excitability results in modulation of the neuronal network. Compared to other methods of neuromodulation, tDCS has widely used in the field of cognitive neuroscience research due to its favorable safety profile, minimal side effects, and cost-effectiveness in comparison with medication-based treatments (64). This is also the reason why tDCS composes the majority of the retrieved NIBS literature. In addition, we found that rTMS did not demonstrate significant therapeutic benefits for ADHD symptoms, the results similar to previous meta-analyses (65).

### 4.2 Selection of targeted brain regions contributes to rehabilitation

More importantly, most all NIBS studies primarily focused on left/right/bilateral dorsal lateral prefrontal cortex (DLPFC) in ADHD (35, 43). For inhibitory control, although none of the NIBS interventions significantly improved inhibitory control compared to sham controls, a trend from pooled effect sizes and SUCRA analysis suggests that tDCS may be beneficial in improving inhibitory control. Children with ADHD exhibit significant delays in executive function abilities compared to typically developing children, with inhibitory control being a key component of executive function. In the majority of studies conducted on tDCS for ADHD, two brain regions have been the primary subjects of investigation: the lateral prefrontal cortex (DLPFC) and the right inferior frontal cortex (rIFC) (35, 53). Research confirms that the right DLPFC serves as a critical neural substrate for inhibitory control, with its modulation enhancing both inhibitory capacity and processing speed in ADHD patients (20). Notably, this NMA reveals a counterintuitive finding: the left DLPFC may constitute a potential therapeutic target for improving inhibitory control and cognitive flexibility. We hypothesize that higher-intensity cathodal stimulation over the left DLPFC could effectively suppress left-sided hyperactivity, thereby disinhibiting right DLPFC activity and augmenting inhibitory control (66). Nevertheless, direct evidence demonstrating left DLPFC stimulation efficacy in enhancing executive functions among ADHD populations remains lacking (67, 68). Future comparative investigations should evaluate the therapeutic efficacy and molecular mechanisms (e.g., dopaminergic signaling, neuroplasticity markers) of differential tDCS montages targeting bilateral DLPFC regions for inhibitory control remediation. Critically, our analysis extends beyond stimulation targets to demonstrate the pivotal role of stimulation polarity in modulating inhibitory processes in ADHD. Anodal tDCS demonstrated the greatest efficacy for enhancing inhibitory control in ADHD populations. Recently, High-Definition transcranial Direct Current Stimulation (HD-tDCS) has been applied to children with ADHD. HD-tDCS offers higher spatial resolution compared to conventional tDCS, enabling more precise stimulation of targeted brain regions (69). However, clinical studies on HD-tDCS for ADHD in children are still limited. Further research is needed to assess its impact on cognitive and social functions in this population, which will help refine personalized treatment strategies for clinical use.

Studies have shown that inhibitory control and working memory are both significantly associated with attention deficit and hyperactivity symptoms, with working memory having the highest correlation with attention deficit and being significantly correlated with brain function (70). For working memory: this study found that a specific stimulation protocol atDCS_F3+ctDCS_F4^*^2 improved working memory performance. Neuroimaging studies have shown reduced brain involvement in tasks involving WM in children with ADHD, particularly in the prefrontal cortex (PFC) and parietal regions, and WM deficits have a direct impact on children's learning, language, math, and social interaction skills (71). Consistent with prior findings demonstrating that tDCS targeting the left DLPFC improves WM in healthy adults and patients with schizophrenia (72), our results further support the efficacy of left DLPFC stimulation for enhancing WM in ADHD patients. Notably, the neurophysiological effects of tDCS exhibit persistence beyond the stimulation period, with improvements in visuospatial WM performance sustained for up to 2 weeks post-stimulation in ADHD. We hypothesize that these lasting effects may arise from the potentiation of practice-dependent synaptic plasticity mediators, such as GABA, glutamate, dopamine, and norepinephrine (67, 73).

This NMA suggested that atDCS_FP2+ctDCS_F3 was significantly better than the sham control in the cognitive flexibility. Cognitive flexibility is the sum of achieving various executive functions that may result from the interaction of specific nodes in the frontal and parietal cortex to adjust behavior. Recent neuroimaging studies have shown that patients with ADHD typically exhibit reduced activity in prefrontal and parietal regions and in the basal ganglia when performing cognitive tasks (74). While DlPFC may have a role in improving cognitive flexibility, similar to inhibitory control mentioned previously.

Further examining inattention and hyperactivity/impulsivity symptoms, although none of the active NIBS treatments produced significant improvements over the sham control group, a trend from pooled effect sizes found that SUCRA analysis suggests that TPS may be improving these symptoms. TPS uses repeated single ultrashort pulses in the ultrasound frequency range to stimulate the brain (75). TPS provides good spatial accuracy and resolution to non-invasively modulate subcortical areas and address cranial attenuation. Studies have identified that the left DLPFC demonstrates significant potential in enhancing attention in children diagnosed with ADHD (54). This phenomenon may be attributable to the observation that TPS instigates augmented neuronal activation and connectivity, not only in the target brain region (i.e., the left DLPFC), but also in other brain regions. However, the application of TPS in clinical interventions remains limited, and further large-scale randomized controlled trials (RCTs) are necessary to validate its efficacy in patients with ADHD.

### 4.3 Strengths and limitations

To the best of our knowledge, this is the first NMA to rank the efficacy of NIBS modes and targeted brains in cognition with ADHD. Additionally, evidence-based clinical decision-making can be enhanced by utilizing NMA, which combines direct and indirect comparisons of trials. This approach provides ranked results that indicate the relative efficacy of each intervention type, aiding in informed decision-making. Notwithstanding, our study does have a few limitations. First, part of NIBS modes was not included in the current NMA, which may have an impact on the completeness of the findings. However, the inclusion has covered common clinical treatments and explains and guides current clinical applications. Second, this NMA used varied ADHD assessment tools and test experiments indifferent studies. This variation is in part due to the lack of a consensus on the outcome measures and the plethora of scales used to assess symptom severity. To mitigate this bias, we specifically selected RCTs that incorporated blinding designs. Additionally, we included as many relevant scales as possible to ensure comprehensive results. However, given the diversity of assessments, it was not feasible to include all interventions in the partial ranking. Therefore, the results of our study should only be applied to the interventions included in the analysis. In addition, we did not perform sensitivity analyses on participants. This is because there are fewer studies involving adolescents and adults to analyze. Participants in this study were free from comorbid psychiatric disorders, though some of the included studies involved individuals with comorbid Conduct Disorder (CD) or Oppositional Defiant Disorder (ODD), both of which are common behavioral disorders in childhood and adolescence and are associated with brain abnormalities. Future research should stratify ADHD patients by age group to further refine our understanding of non-invasive brain stimulation (NIBS) effects on cognitive function.

## 5 Conclusions

This NMA comprehensively evaluated the efficacy and acceptability of NIBS techniques for enhancing cognitive function in individuals with ADHD. Our findings indicate that dual-tDCS and a-tDCS may be considered among the preferred NIBS interventions for improving cognitive function in ADHD, although further confirmatory trials are warranted. Specifically, atDCS_F3 + ctDCS_FP2 improved cognitive flexibility; while atDCS_F3 + ctDCS_F4 enhanced working memory. For inhibitory control, both dual-tDCS and a-tDCS demonstrated superior efficacy relative to rTMS. However, further research is needed to investigate TPS for improving attention and impulsivity. The left DLPFC was the recommended region for improving cognitive function in ADHD, and the right IFC proved limited benefit. Future meta-analyses should specifically examine the role of stimulation intensity. Based on these findings, future research should focus on optimizing existing NIBS protocols and exploring novel stimulation modalities.

## Data Availability

The original contributions presented in the study are included in the article/Supplementary material, further inquiries can be directed to the corresponding authors.
