# Supplementary material for: Comparative efficacy of non-invasive brain stimulation for attention-deficit/hyperactivity disorder: a systematic review and network meta-analysis
Source: Front Neurol. 2025 Oct 2;16:1650154. doi: 10.3389/fneur.2025.1650154 (PMC12527846; doi:10.3389/fneur.2025.1650154)
Supplement: Supplementary file 1 [file Table_1.docx]

Comparative efficacy of Non-Invasive Brain Stimulation for Attention-Deficit/Hyperactivity Disorder: A Systematic Review and Network Meta-Analysis

| **Table of Contents** | | |
| --- | --- | --- |
| Title | Content | page |
| Table S1 | The comparisons between this review and the original PROSPERO protocol |  |
| Table S2 | PRISMA Checklist of Items to Include When Reporting a Systematic Review Involving a Meta-analysis |  |
| Table S3 | Criteria for Inclusion and Exclusion |  |
| Table S4 | Literature Search Strategy |  |
| Table S5 | Sociodemographic and clinical characteristics of the included studies |  |
| Table S6 | Intervention-related features of the included studies |  |
| Table S7 | Study design and measurement-related characteristics |  |

Table S1. The comparisons between this review and the original PROSPERO protocol

| The section | Network meta-analysis | PROSPERO protocol |
| --- | --- | --- |
| Population | participants with a diagnosis of Attention-Deficit/Hyperactivity Disorder (ADHD) | Adults and Children with Attention Deficit/Hyperactivity Disorder |
| Intervention | Non-invasive brain stimulation (NIBS) modes, including rTMS, tDCS, and other variants | Non-invasive Brain Stimulation (NIBS) is a broad term that includes all technologies that, through specific mechanisms of action, can induce Brain Stimulation and/or Brain Neuromodulation, for treatment of neuropsychiatric disorders. Also, they do not require sedation or anesthesia. Most frequently NIBS technologies applied in clinical settings include Transcranial Magnetic Stimulation (TMS) - repetitive TMS (rTMS), deep TMS (dTMS) and Theta Burst Stimulation (TBS) - and Transcranial Direct Current Stimulation (tDCS). Other examples include Transcranial Alternating Current Stimulation (tACS), Transcranial Random Noise Stimulation (tRNS) and Trigeminal Nerve Stimulation (TNS). |
| Comparison | sham stimulation or placebo therapy | Sham NIBS, other active NIBS or standard treatment for ADHD. |
| Outcomes | standardized tests for assessment of attentional and executive function, such as inhibitory control, working memory, cognitive flexibility, inattention, hyperactivity and impulsivity | inhibitory control, working memory, and cognitive flexibility |
| Study design | Systematic Review and Network Meta-Analysis | Systematic Review and Network Meta-Analysis |

Table S2. PRISMA Checklist of Items to Include When Reporting a Systematic Review Involving a Meta-analysis

| **Section/Topic** | **Item #** | **Checklist Item** | **Reported on Page #** |
| --- | --- | --- | --- |
| **TITLE** |  |  |  |
| Title | 1 | Comparative efficacy of Non-Invasive Brain Stimulation for Attention-Deficit/Hyperactivity Disorder: A Systematic Review and Network Meta-Analysis |  |
|  |  |  |  |
| **ABSTRACT** |  |  |  |
| Structured summary | 2 | Provide a structured summary including, as applicable:  **Background:** main objectives  **Methods:** data sources; study eligibility criteria, participants, and interventions; study appraisal; and *synthesis methods, such as network meta-analysis.*  **Results:** number of studies and participants identified; summary estimates with corresponding confidence/credible intervals; *treatment rankings may also be discussed. Authors may choose to summarize pairwise comparisons against a chosen treatment included in their analyses for brevity.*  **Discussion/Conclusions:** limitations; conclusions and implications of findings.  **Other:** systematic review registration number with registry name. |  |
|  |  |  |  |
| **INTRODUCTION** |  |  |  |
| Rationale | 3 | Describe the rationale for the review in the context of what is already known*, including mention of why a network meta-analysis has been conducted.* |  |
| Objectives | 4 | Provide an explicit statement of questions being addressed, with reference to participants, interventions, comparisons, outcomes, and study design (PICOS). |  |
|  |  |  |  |
| **METHODS** |  |  |  |
| Protocol and registration | 5 | Indicate whether a review protocol exists and if and where it can be accessed (e.g., Web address); and, if available, provide registration information, including registration number. |  |
| Eligibility criteria | 6 | Specify study characteristics (e.g., PICOS, length of follow-up) and report characteristics (e.g., years considered, language, publication status) used as criteria for eligibility, giving rationale. *Clearly describe eligible treatments included in the treatment network, and note whether any have been clustered or merged into the same node (with justification).* |  |
| Information sources | 7 | Describe all information sources (e.g., databases with dates of coverage, contact with study authors to identify additional studies) in the search and date last searched. |  |
| Search | 8 | Present full electronic search strategy for at least one database, including any limits used, such that it could be repeated. |  |
| Study selection | 9 | State the process for selecting studies (i.e., screening, eligibility, included in systematic review, and, if applicable, included in the meta-analysis). |  |
| Data collection process | 10 | Describe method of data extraction from reports (e.g., piloted forms, independently, in duplicate) and any processes for obtaining and confirming data from investigators. |  |
| Data items | 11 | List and define all variables for which data were sought (e.g., PICOS, funding sources) and any assumptions and simplifications made. |  |
| **Geometry of the network** | **S1** | Describe methods used to explore the geometry of the treatment network under study and potential biases related to it. This should include how the evidence base has been graphically summarized for presentation, and what characteristics were compiled and used to describe the evidence base to readers. |  |
| Risk of bias within individual studies | 12 | Describe methods used for assessing risk of bias of individual studies (including specification of whether this was done at the study or outcome level), and how this information is to be used in any data synthesis. |  |
| Summary measures | 13 | State the principal summary measures (e.g., risk ratio, difference in means). *Also describe the use of additional summary measures assessed, such as treatment rankings and surface under the cumulative ranking curve (SUCRA) values, as well as modified approaches used to present summary findings from meta-analyses.* |  |
| Planned methods of analysis | 14 | Describe the methods of handling data and combining results of studies for each network meta-analysis. This should include, but not be limited to:   - *Handling of multi-arm trials;* - *Selection of variance structure;* - *Selection of prior distributions in Bayesian analyses; and* - *Assessment of model fit.* |  |
| **Assessment of Inconsistency** | **S2** | Describe the statistical methods used to evaluate the agreement of direct and indirect evidence in the treatment network(s) studied. Describe efforts taken to address its presence when found. |  |
| Risk of bias across studies | 15 | Specify any assessment of risk of bias that may affect the cumulative evidence (e.g., publication bias, selective reporting within studies). |  |
| Additional analyses | 16 | Describe methods of additional analyses if done, indicating which were pre-specified. This may include, but not be limited to, the following:   - Sensitivity or subgroup analyses; - Meta-regression analyses; - *Alternative formulations of the treatment network; and* - *Use of alternative prior distributions for Bayesian analyses (if applicable).* |  |
|  |  |  |  |
| **RESULTS†** |  |  |  |
| Study selection | 17 | Give numbers of studies screened, assessed for eligibility, and included in the review, with reasons for exclusions at each stage, ideally with a flow diagram. |  |
| **Presentation of network structure** | **S3** | Provide a network graph of the included studies to enable visualization of the geometry of the treatment network. |  |
| **Summary of network geometry** | **S4** | Provide a brief overview of characteristics of the treatment network. This may include commentary on the abundance of trials and randomized patients for the different interventions and pairwise comparisons in the network, gaps of evidence in the treatment network, and potential biases reflected by the network structure. |  |
| Study characteristics | 18 | For each study, present characteristics for which data were extracted (e.g., study size, PICOS, follow-up period) and provide the citations. |  |
| Risk of bias within studies | 19 | Present data on risk of bias of each study and, if available, any outcome level assessment. |  |
| Results of individual studies | 20 | For all outcomes considered (benefits or harms), present, for each study: 1) simple summary data for each intervention group, and 2) effect estimates and confidence intervals. *Modified approaches may be needed to deal with information from larger networks.* |  |
| Synthesis of results | 21 | Present results of each meta-analysis done, including confidence/credible intervals. *In larger networks, authors may focus on comparisons versus a particular comparator (e.g. placebo or standard care), with full findings presented in an appendix. League tables and forest plots may be considered to summarize pairwise comparisons.* If additional summary measures were explored (such as treatment rankings), these should also be presented. |  |
| **Exploration for inconsistency** | **S5** | Describe results from investigations of inconsistency. This may include such information as measures of model fit to compare consistency and inconsistency models, *P* values from statistical tests, or summary of inconsistency estimates from different parts of the treatment network. |  |
| Risk of bias across studies | 22 | Present results of any assessment of risk of bias across studies for the evidence base being studied. |  |
| Results of additional analyses | 23 | Give results of additional analyses, if done (e.g., sensitivity or subgroup analyses, meta-regression analyses*, alternative network geometries studied, alternative choice of prior distributions for Bayesian analyses,* and so forth). |  |
|  |  |  |  |
| **DISCUSSION** |  |  |  |
| Summary of evidence | 24 | Summarize the main findings, including the strength of evidence for each main outcome; consider their relevance to key groups (e.g., healthcare providers, users, and policy-makers). |  |
| Limitations | 25 | Discuss limitations at study and outcome level (e.g., risk of bias), and at review level (e.g., incomplete retrieval of identified research, reporting bias). *Comment on the validity of the assumptions, such as transitivity and consistency. Comment on any concerns regarding network geometry (e.g., avoidance of certain comparisons).* |  |
| Conclusions | 26 | Provide a general interpretation of the results in the context of other evidence, and implications for future research. |  |
|  |  |  |  |
| **FUNDING** |  |  |  |
| Funding | 27 | Describe sources of funding for the systematic review and other support (e.g., supply of data); role of funders for the systematic review. This should also include information regarding whether funding has been received from manufacturers of treatments in the network and/or whether some of the authors are content experts with professional conflicts of interest that could affect use of treatments in the network. |  |

PICOS = population, intervention, comparators, outcomes, study design.

* Text in italics indicate S wording specific to reporting of network meta-analyses that has been added to guidance from the PRISMA statement.

† Authors may wish to plan for use of appendices to present all relevant information in full detail for items in this section.

Table S3. Criteria for Inclusion and Exclusion

| **Inclusion Criteria** | 1. Participants: participants with a diagnosis of ADHD; 2. Intervention: Non-invasive brain stimulation (NIBS) modes, including rTMS, tDCS, and other variants; 3. Comparison: sham stimulation or placebo therapy; 4. Outcome: standardized tests for assessment of attentional and executive function, such as inhibitory control, working memory, cognitive flexibility, inattention, hyperactivity and impulsivity; 5. Study design: clinical randomized controlled trials (RCTs) with human participants. |
| --- | --- |
| **Exclusion Criteria** | 1. they enrolled non-ADHD subjects; 2. used interventions irrelevant to non-invasive brain stimulation; 3. control groups were not appropriate, e.g., in terms of mismatched age, sex, or severity of disease between groups; 4. the data was incomplete; 5. they were low-quality study types including case series or reports, conference papers, and non-peer-revied articles. |

Table S4. Literature Search Strategy

| **Table S2.Literature Search Strategy** | |
| --- | --- |
| **Pubmed** | #1 “Attention Deficit Disorder with Hyperactivity” OR “ADHD” OR “ADDH” OR “Attention Deficit Disorders with Hyperactivity” OR “Attention Deficit Hyperactivity Disorders” OR “Attention Deficit Hyperactivity Disorder” OR “Attention Deficit-Hyperactivity Disorder” OR “Attention Deficit-Hyperactivity Disorders” OR “Deficit-Hyperactivity Disorder, Attention” OR “Deficit-Hyperactivity Disorders, Attention” OR “Disorder, Attention Deficit-Hyperactivity” OR “Disorders, Attention Deficit-Hyperactivity” OR “Hyperkinetic Syndrome” OR “Syndromes, Hyperkinetic” OR “Attention Deficit Disorder” OR “Attention Deficit Disorders” OR “Deficit Disorder, Attention” OR “Deficit Disorders, Attention” OR “Disorder, Attention Deficit” OR “Disorders, Attention Deficit” OR “Brain Dysfunction, Minimal” OR “Dysfunction, Minimal Brain” OR “Minimal Brain Dysfunction”  #2“non-invasive brain stimulation” OR “noninvasive brain stimulation”  #3 “transcranial direct current stimulation” OR “tDCS” OR “Anodal Stimulation Transcranial Direct Current Stimulation” OR “Anodal Stimulation tDCS” OR “Anodal Stimulation tDCSs” OR “Stimulation tDCS, Anodal” OR “Stimulation tDCSs, Anodal” OR “tDCS, Anodal Stimulation” OR “tDCSs, Anodal Stimulation” OR “Cathodal Stimulation Transcranial Direct Current Stimulation” OR “Cathodal Stimulation tDCS” OR “Cathodal Stimulation tDCSs” OR “Stimulation tDCS, Cathodal” OR “Stimulation tDCSs, Cathodal” OR “tDCS, Cathodal Stimulation” OR “tDCSs, Cathodal Stimulation” OR “Transcranial Alternating Current Stimulation” OR “Transcranial Random Noise Stimulation” OR “Repetitive Transcranial Electrical Stimulation” OR “Transcranial Electrical Stimulation” OR “Electrical Stimulations, Transcranial” OR “Electrical Stimulation, Transcranial” OR “Stimulations, Transcranial Electrical” OR “Stimulation, Transcranial Electrical” OR “Transcranial Electrical Stimulations”  #4 “Transcranial Magnetic Stimulation” OR “Magnetic Stimulations, Transcranial” OR “Magnetic Stimulation, Transcranial” OR “Stimulations, Transcranial Magnetic” OR “Stimulation, Transcranial Magnetic” OR “Transcranial Magnetic Stimulations” OR “Transcranial Magnetic Stimulation, Paired Pulse” OR “Transcranial Magnetic Stimulation, Repetitive” OR “Transcranial Magnetic Stimulation, Single Pulse” OR “transcranial pulse stimulation”  #5 “Ultrasonic Therapy” OR “Therapies, Ultrasonic” OR “Ultrasonic Therapies” OR “Ultrasound Therapy” OR “Therapies, Ultrasound” OR “Therapy, Ultrasound” OR “Ultrasound Therapies” OR “Therapeutic Ultrasound” OR “Ultrasound, Therapeutic” OR “Therapy, Ultrasonic”  #6 #2 OR #3 OR #4 OR #5  #7“Cognition” OR “Cognitions” OR “Cognitive Function” OR “Cognitive Functions” OR “Function, Cognitive” OR “Functions, Cognitive” OR “Insight” OR “Insights”  #8 “Cognitive Dysfunction” OR “Cognitive Dysfunctions” OR “Dysfunction, Cognitive” OR “Dysfunctions, Cognitive” OR “Cognitive Disorder” OR “Cognitive Disorders” OR “Disorder, Cognitive” OR “Disorders, Cognitive” OR “Cognitive Impairments” OR “Cognitive Impairment” OR “Impairment, Cognitive” OR “Impairments, Cognitive” OR “Mild Cognitive Impairment” OR “Cognitive Impairment, Mild” OR “Cognitive Impairments, Mild” OR “Impairment, Mild Cognitive” OR “Impairments, Mild Cognitive” OR “Mild Cognitive Impairments” OR “Cognitive Decline” OR “Cognitive Declines” OR “Decline, Cognitive” OR “Declines, Cognitive” OR “Mental Deterioration” OR “Deterioration, Mental” OR “Deteriorations, Mental” OR “Mental Deteriorations”  #9 #7 OR #8  #10 #1 AND #6 AND #9 |
| **Web of Science** | #1 ((((((((((((((((((TS=(Attention Deficit Disorder with Hyperactivity)) OR TS=(ADHD)) OR TS=(Attention Deficit Disorders with Hyperactivity)) OR TS=(Attention Deficit Hyperactivity Disorders)) OR TS=(Attention Deficit Hyperactivity Disorder)) OR TS=(Attention Deficit-Hyperactivity Disorder)) OR TS=(Attention Deficit-Hyperactivity Disorders)) OR TS=(Hyperkinetic Syndrome)) OR TS=(Attention Deficit Disorder)) OR TS=(Attention Deficit Disorders)) OR TS=(Minimal Brain Dysfunction)) OR TS=(Impulsive Behavior)) OR TS=(Impulsive Behaviors)) OR TS=(Impulsivity)) OR TS=(Impulsivities)) OR TS=(hyperkinetic disorder)) OR TS=(attention-deficit hyperactivity disorder)) OR TS=(inattention)) OR TS=(hyperactivity)  #2 (((((((((((((((((((((((((TS=(Transcranial Magnetic Stimulation)) OR TS=(Magnetic Stimulations, Transcranial)) OR TS=(Magnetic Stimulation, Transcranial)) OR TS=(Transcranial Magnetic Stimulations)) OR TS=(Transcranial Magnetic Stimulation, Paired Pulse)) OR TS=(Transcranial Magnetic Stimulation, Repetitive)) OR TS=(Transcranial Magnetic Stimulation, Single Pulse)) OR TS=(Transcranial Direct Current Stimulation)) OR TS=(tDCS)) OR TS=(Anodal Stimulation Transcranial Direct Current Stimulation)) OR TS=(Anodal Stimulation tDCS)) OR TS=(Anodal Stimulation tDCSs)) OR TS=(Cathodal Stimulation Transcranial Direct Current Stimulation)) OR TS=(Cathodal Stimulation tDCS)) OR TS=(Cathodal Stimulation tDCSs)) OR TS=(Transcranial Alternating Current Stimulation)) OR TS=(Transcranial Random Noise Stimulation)) OR TS=(Repetitive Transcranial Electrical Stimulation)) OR TS=(Transcranial Electrical Stimulation)) OR TS=(Transcranial Electrical Stimulations)) OR TS=(Transcranial Magnetic Stimulation)) OR TS=(Transcranial Magnetic Stimulations)) OR TS=(Transcranial Magnetic Stimulation, Paired Pulse)) OR TS=(Transcranial Magnetic Stimulation, Repetitive)) OR TS=(Transcranial Magnetic Stimulation, Single Pulse)) OR TS=(non-invasive brain stimulation)  #3 (((((((((((((((((((TS=(Cognitive Dysfunction)) OR TS=(Cognitive Dysfunctions)) OR TS=(Cognitive Disorder)) OR TS=(Cognitive Disorders)) OR TS=(Cognitive Impairments)) OR TS=(Cognitive Impairment)) OR TS=(Mild Cognitive Impairment)) OR TS=(Mild Cognitive Impairments)) OR TS=(Cognitive Decline)) OR TS=(Cognitive Declines)) OR TS=(Mental Deterioration)) OR TS=(Mental Deteriorations)) OR TS=(Cognition)) OR TS=(Cognitions)) OR TS=(Cognitive Function)) OR TS=(Cognitive Functions)) OR TS=(Insight)) OR TS=(Insights)) OR TS=(cognitive impairment)) OR TS=(cognitive dysfunction)  #4 #1 AND #2 AND #3 |
| **Cochrane** | #1 (ADHD or Attention Deficit Disorders with Hyperactivity or Attention Deficit Hyperactivity Disorders or Attention Deficit Hyperactivity Disorder or Attention Deficit-Hyperactivity Disorder or Attention Deficit-Hyperactivity Disorders or Hyperkinetic Syndrome or Attention Deficit Disorder or Attention Deficit Disorders or Minimal Brain Dysfunction)  #3 (Impulsive Behaviors or Impulsivity or Impulsivities)  #4 (hyperkinetic disorder or attention-deficit hyperactivity disorder or inattention or hyperactivity)  #5 #1 OR #2 OR #3 OR #4  #6 (Transcranial Magnetic Stimulation or Magnetic Stimulations, Transcranial or Magnetic Stimulation, Transcranial or Transcranial Magnetic Stimulations or Transcranial Magnetic Stimulation, Paired Pulse or Transcranial Magnetic Stimulation, Repetitive or Transcranial Magnetic Stimulation, Single Pulse)  #7 ( Transcranial Direct Current Stimulation or tDCS or Anodal Stimulation Transcranial Direct Current Stimulation or Anodal Stimulation tDCS or Anodal Stimulation tDCSs or Cathodal Stimulation Transcranial Direct Current Stimulation or Cathodal Stimulation tDCS or Cathodal Stimulation tDCSs or Transcranial Alternating Current Stimulation or Transcranial Random Noise Stimulation or Repetitive Transcranial Electrical Stimulation or Transcranial Electrical Stimulation or Transcranial Electrical Stimulations)  #8 (non-invasive brain stimulation)  #9 #6 OR #7 OR #8  #10(Cognitive Dysfunctions or Cognitive Disorder or Cognitive Disorders or Cognitive Impairments or Cognitive Impairment or Mild Cognitive Impairment or Mild Cognitive Impairments or Cognitive Decline or Cognitive Declines or Mental Deterioration or Mental Deteriorations)  #11 ( Cognition or Cognitions or Cognitive Function or Cognitive Functions or Insight or Insights)  #12 (cognitive impairment or cognitive dysfunction)  #13 #10 OR #11 OR #12  #14 #5 AND #9 AND #13 |
| **Embase** | #1 'attention deficit disorder with hyperactivity'/exp OR 'attention deficit disorder with hyperactivity' OR 'adhd'/exp OR 'adhd' OR 'attention deficit disorders with hyperactivity' OR 'attention deficit hyperactivity disorders' OR 'attention deficit hyperactivity disorder'/exp OR 'attention deficit hyperactivity disorder' OR 'attention deficit-hyperactivity disorder'/exp OR 'attention deficit-hyperactivity disorder' OR 'attention deficit-hyperactivity disorders' OR 'hyperkinetic syndrome'/exp OR 'hyperkinetic syndrome' OR 'attention deficit disorder'/exp OR 'attention deficit disorder' OR 'attention deficit disorders' OR 'minimal brain dysfunction'/exp OR 'minimal brain dysfunction'  #2 'impulsive behavior'/exp OR 'impulsive behavior' OR 'impulsive behaviors' OR 'impulsivity'/exp OR 'impulsivity' OR 'impulsivities'  #3 'hyperkinetic disorder'/exp OR 'hyperkinetic disorder' OR 'attention-deficit hyperactivity disorder'/exp OR 'attention-deficit hyperactivity disorder' OR 'inattention'/exp OR 'inattention' OR 'hyperactivity'/exp OR 'hyperactivity'  #4 'transcranial magnetic stimulation'/exp OR 'transcranial magnetic stimulation' OR 'magnetic stimulations, transcranial' OR 'magnetic stimulation, transcranial'/exp OR 'magnetic stimulation, transcranial' OR 'transcranial magnetic stimulations' OR 'transcranial magnetic stimulation, paired pulse' OR 'transcranial magnetic stimulation, repetitive'/exp OR 'transcranial magnetic stimulation, repetitive' OR 'transcranial magnetic stimulation, single pulse'  #5 'transcranial direct current stimulation' OR 'tdcs' OR 'anodal stimulation transcranial direct current stimulation' OR 'anodal stimulation tdcs' OR 'anodal stimulation tdcss' OR 'cathodal stimulation transcranial direct current stimulation' OR 'cathodal stimulation tdcs' OR 'cathodal stimulation tdcss' OR 'transcranial alternating current stimulation' OR 'transcranial random noise stimulation' OR 'repetitive transcranial electrical stimulation' OR 'transcranial electrical stimulation' OR 'transcranial electrical stimulations'  #6 #1 OR #2 OR #3  #7 'transcranial magnetic stimulation'/exp OR 'transcranial magnetic stimulation' OR 'transcranial magnetic stimulations' OR 'transcranial magnetic stimulation, paired pulse' OR 'transcranial magnetic stimulation, repetitive'/exp OR 'transcranial magnetic stimulation, repetitive' OR 'transcranial magnetic stimulation, single pulse'  #8 'non-invasive brain stimulation' OR ('non invasive' AND ('brain'/exp OR brain) AND ('stimulation'/exp OR stimulation))  #9 #4 OR #5 OR #7 OR #8  #10 'cognitive dysfunctions' OR 'cognitive disorder'/exp OR 'cognitive disorder' OR 'cognitive disorders'/exp OR 'cognitive disorders' OR 'cognitive impairments' OR 'cognitive impairment'/exp OR 'cognitive impairment' OR 'mild cognitive impairment'/exp OR 'mild cognitive impairment' OR 'mild cognitive impairments' OR 'cognitive decline'/exp OR 'cognitive decline' OR 'cognitive declines' OR 'mental deterioration'/exp OR 'mental deterioration' OR 'mental deteriorations'  #11 'cognitions' OR 'cognitive function'/exp OR 'cognitive function' OR 'cognitive functions' OR 'insight'/exp OR 'insight' OR 'insights' OR 'cognitive impairment'/exp OR 'cognitive impairment' OR 'cognitive dysfunction'/exp OR 'cognitive dysfunction'  #12 #10 OR #11  #13 #6 AND #9 AND #12 |
| **China Knowledge Network (CNKI)** | #1 (Title/Abstract: "Attention Deficit Hyperactivity Disorder" [Exact]) OR (Title/Abstract: "Attention Deficit and Disruptive Behavior Disorders" [Exact]) OR (Title/Abstract: "Impulsive Behavior" [Exact]) OR (Title/Abstract: "Autism Spectrum Disorder" [Exact])  #2 (Title/Abstract: "Non-invasive Brain Stimulation" [Exact]) OR (Title/Abstract: "Transcranial Direct Current Stimulation" [Exact]) OR (Title/Abstract: "Repetitive Transcranial Magnetic Stimulation" [Exact]) OR (Title/Abstract: "Transcranial Pulsed Stimulation" [Exact]) OR (Title/Abstract: "Ultrasound" [Exact]) OR (Title/Abstract: "Cognitive Impairment" [Exact]) OR (Title/Abstract: "Cognition" [Exact])  #3 #1 AND #2 |
| **Wanfang database** | #1 (Title/Keyword: "Attention Deficit Hyperactivity Disorder") OR (Title/Keyword: "Attention Deficit and Disruptive Behavior Disorders") OR (Title/Keyword: "Impulsive Behavior") OR (Title/Keyword: "Autism Spectrum Disorder")  #2 (Title/Keyword: "Non-invasive Brain Stimulation") OR (Title/Keyword: "Transcranial Direct Current Stimulation") OR (Title/Keyword: "Repetitive Transcranial Magnetic Stimulation") OR (Title/Keyword: "Transcranial Pulsed Stimulation") OR (Title/Keyword: "Ultrasound") OR (Title/Keyword: "Cognitive Impairment") OR (Title/Keyword: "Cognition")  #3 #1 AND #2 |
| **Chongqing Weipu (VIP)** | #1 (Title/Abstract/Keyword: "Attention Deficit Hyperactivity Disorder") OR (Title/Abstract/Keyword: "Attention Deficit and Disruptive Behavior Disorders") OR (Title/Abstract/Keyword: "Impulsive Behavior") OR (Title/Abstract/Keyword: "Autism Spectrum Disorder")  #2 (Title/Abstract/Keyword: "Non-invasive Brain Stimulation") OR (Title/Abstract/Keyword: "Transcranial Direct Current Stimulation") OR (Title/Abstract/Keyword: "Repetitive Transcranial Magnetic Stimulation") OR (Title/Abstract/Keyword: "Transcranial Pulsed Stimulation") OR (Title/Abstract/Keyword: "Ultrasound") OR (Title/Abstract/Keyword: "Cognitive Impairment") OR (Title/Abstract/Keyword: "Cognition")  #3 #1 AND #2 |
|  |  |

Table S5. Sociodemographic and clinical characteristics of the included studies

| study | first language | number of participants | | | | mean age(years, SD) | | | | age distribution（children, adolescents, adults, or mixed） | | | sex(male: female) | | | |
| --- | --- | --- | --- | --- | --- | --- | --- | --- | --- | --- | --- | --- | --- | --- | --- | --- |
|  |  | Exp/Exp1 | Sham/Exp2 | | Ctr/Exp3 | Exp/Exp1 | Sham/Exp2 | | Ctr | Exp/Exp1 | Sham/Exp2 | Ctr | Exp/Exp1 | Sham/Exp2 | | Ctr |
| Cosmo et al.2015 | English | 30 | 30 | |  | 31.83(11.55) | 32.67(10.37) | |  | adults | |  | 17:13 | 18:12 | |  |
| Breitling et al.2016 | English | 21 |  | | 21 | 14.33(NM) |  | | 14.24(NM) | adolescents | |  | 21:0 |  | | 21:0 |
| Cachoeira et al.2017 | English | 9 | 8 | |  | 31(6.17) | 33.75(3.65) | |  | adults | |  | 4:5 | 4:4 | |  |
| Heng et al.2017 | Chinese | 30 |  | | 30 | 8.6(NM) | | | | children | |  |  | | | |
| Soff et al.2017 | English | 15 | | |  | 14.2(1.2) | | |  | adolescents | |  |  | | |  |
| Allenby et al.2018 | English | 37 | | |  | 31.7(NM) | | |  | adults | |  |  | | |  |
| Cao et al.2018 | English | 21 | 20 | | 19 | 8.50(2.20) | 8.36(2.46) | | 9.22(2.39) | children, adolescents | | | 15:6 | 18:2 | | 16:3 |
| Paz et al.2018 | English | 9 | 13 | |  | 32.11(6.47) | 30.85(6.82) | |  | adults | |  | 6:3 | 8:5 | |  |
| Soltaninejad et al.2019 | English | 20 | | |  | 16.1(NM) | | |  | adolescents | |  |  | | |  |
| Alyagon et al.2020 | English | 15 | 14 | | 14 | 26.62 (0.66) | 27.64 (1.58) | | 26.13(0.59) | adults | | | 13:2 | 11:3 | | 10:4 |
| Breitling et al.2020 | English | 15 | | |  | 13.3(1.9) | 13.3(1.8) | |  | children, adolescents | |  | 12:2 | 13:2 | |  |
| Nejati et al.2020 | English | 15 | 10 | |  | 10(2.3) | 9(1.8) | |  | children, adolescents | |  | 15:0 | 5:5 | |  |
| Salehinejad et al.2020 | English | 17 | | |  | 9.53(1.50) | | |  | children | |  |  | | |  |
| Breitling-Ziegler et al.2021 | English | 20 | 13 | | 13 | 10-17 | 13.54(1.45) | | 14.08(2.10) | children, adolescents | |  | 15:5 | 11:2 | | 10:3 |
| Westwood et al.2021 | English | 24 | 26 | |  | 13.05(1.98) | 14.23(2.06) | |  | children, adolescents | |  | 24:0 | 26:0 | |  |
| Amouzadeh et al.2022 | English | 15 | 15 | | 15 | 11.62(0.35) | 11.60(0.34) | | 11.6(0.34) | children, adolescents | | | 10:5 | 10:5 | | 10:5 |
| Barham et al.2022 | English | 11 | 11 | |  | 22.45(3) | 21.72(2.61) | |  | adults | |  | 5:6 | 2:9 | |  |
| Dakwar-Kawar et al.2022 | English | 19 | | |  | 6-12 | | |  | children | |  |  | | |  |
| Kannen et al.2022 | English | 19 | | |  | 27.95 (8.57) | | |  | adults | |  |  | | |  |
| Klomjai et al.2022 | English | 11 | | |  | 8.55(0.65) | | |  | children, adolescents | |  |  | | |  |
| Leffa et al.2022 | English | 32 | 32 | |  | 38.2 (10.3) | 38.4 (9.1) | |  | adults | |  | 13:19 | 21:11 | |  |
| Liang et al.2022 | Chinese | 23 | 23 | | 23 | 7.45(2.44) | 8.26(0.71) | | 7.63(1.59) | children | | | 18:5 | 19:4 | | 18:5 |
| Makkar et al.2022 | English | 30 | 31 | |  | 12.80(1.75) | 12.81(2.01） | |  | children | |  | 27:3 | 22:9 | |  |
| Nagy et al.2022 | English | 30 | 30 | |  | 8.7（1.76） | 8.47（1.7） | |  | children | |  | 24:6 | 22:8 | |  |
| Nejati et al.2022 | English | 24 | 25 | |  | 9.25 (1.53) | 9.35 (1.12) | |  | children | |  | 16:8 | 15:10 | |  |
| Salehinejad et al.2022 | English | 22 | | |  | 8.86(1.80) | | |  | children | |  |  | | |  |
| Westwood et al.2022 | English | 10 | 13 | |  | 10-18 | | |  | mixed | |  | 10:0 | 13:0 | |  |
| Nejati et al.2023 | English | 23 | | |  | 10.09(2.02) | | |  | children, adolescents | |  |  | | |  |
| Bian et al.2024 | Chinese | 103 |  | | 99 | 8.00±52 |  | | 7.68±2.41 | children |  | children | 86:17 |  | | 84:15 |
| Cheung et al.2024 | English | 17 | 15 | |  | 12.8 (1.51) | 13.3 (1.34) | |  | adolescents | |  | 13:4 | 12:3 | |  |
| Estaji et al.2024 | English | 24 | |  | | 9.16(1.57) | |  | | children | | | 18:6 | |  | |
| Guimarães et al.2024 | English | 15 | |  | | 11.2 (3.0) | |  | | children, adolescents | | | 10:5 | |  | |
| Kannen et al.2024 | English | 15 | |  | | 32.53(11.07) | |  | | adults | | | 11:4 | |  | |
| Nejati et al.2024 | English | 18 | |  | | 9.89 (1.90) | |  | | children | | | 12:6 | |  | |
| Tian et al.2024 | English | 62 | 64 | | 64 | 9.50 (8.00,11.00) | 10.00 (8.00,11.00) | | 10.00 (8.25,11.00) | children | | | 48:14 | 49:15 | | 45:19 |
| Wang et al.2024 | English | 17 |  | | 18 | 8.45(1.53) |  | | 8.23(1.42) | children |  | children | 12:5 |  | | 11:7 |
| Yang et al.2024 | Chinese | 34 |  | | 34 | 9.14±1. 31 |  | | 9.21±1. 28 | children |  | children | 15:19 |  | | 18:16 |

Table S6. Intervention-related features of the included studies

| study | parametres of NIBS | | | | | Characteristics of NIBS therapy | | | | |
| --- | --- | --- | --- | --- | --- | --- | --- | --- | --- | --- |
|  | type of NIBS | Type of stimulation | Frequncy/intensity of stimulation | Targeted brain  region | Stimulation  position/co-ordinate | Period of each therapy condition | Number of sessions in each therapy condition | Frequency | Duration of  each session |  |
| Cosmo et al.2015 | tDCS | Anodal+Cathodal | 1mA | Bi-DLPFC | Anodal:F3; cathodal:F4 | 1 time | 1 | 1 | 20min |  |
| Breitling et al.2016 | tDCS | Anodal | 1mA | R-IFG | Anodal:F8; cathodal:P7 | 3 times | 3 | 1 | 20min |  |
| Cachoeira et al.2017 | tDCS | Anodal+Cathodal | 2mA | Bi-DLPFC | Anodal:F4; cathodal:F3 | 5days | 5 | 1 | 20min |  |
| Heng et al.2017 | rTMS | HF | 10Hz | R-DLPFC | NM | 8w | 40 | 5/w | 30min |  |
| Soff et al.2017 | tDCS | Anodal | 1mA | L-DLPFC | Anodal:F3  cathodal: vertex | 2 times | 5 active+ 5 sham | 5/w | 20min |  |
| Allenby et al.2018 | tDCS | Anodal | 2mA | L-DLPFC | Anodal:F3; cathodal: Fp2 | 1w | 3 | 3/w | 20min |  |
| Cao et al.2018 | rTMS | HF | 10Hz | R-DLPFC | NM | 6w | 30 | 5/w | 25min |  |
| Paz et al.2018 | dTMS | HF | 18Hz | Bi-DLPFC | NM | 4w | 28 | 7/w | NM |  |
| Soltaninejad et al.2019 | tDCS | Anodal/Cathodal | 1.5mA | R-DLPFC | Anodal/cathodal:F3; cathodal/Anodal:Fp2 | 3 times | 3 | 1 | 15min |  |
| Alyagon et al.2020 | rTMS | HF | 18Hz | R-PFC | NM | 3w | 15 | 5/w | NM |  |
| Breitling et al.2020 | HD-tDCS | Anodal | 1mA(tDCs)/0.5mA(HD-tDCS) | R-IFG | Anodal:F8; cathodal:Fp2 | 3 times | 3 | 1 | 20min |  |
| Nejati et al.2020 | tDCS | Anodal/Cathodal | 1mA | Exp1:Bi-DLPFC Exp2:DLPFC/OFC | Exp1：anodal F3 cathodal F4； Exp2：anodal F3  cathodal Fp2  and  cathodal F3   anodal Fp2 | 2 times/ 3 times | 2/3 | 1 | 15min |  |
| Salehinejad et al.2020 | tDCS | Anodal | 1mA | R-PPC | Anodal:P4; cathodal: contralateral shoulder | 2 times | 1 active+1 sham | 1 | 15min |  |
| Breitling-Ziegler et al.2021 | HD-tDCS | Anodal | 0.5mA or 0.25mA | R-IFG | Anodal: center of IFG; cathodal: surrounded by four cathodes in a distance of 4 cm | 5 times | 5 | 1 | 20min |  |
| Westwood et al.2021 | tDCS | Anodal | 1mA | R-IFC | Anodal:F8; cathodal:Fp1 | 3w | 15 | 5/w | 20min |  |
| Amouzadeh et al.2022 | tACS | Anodal+Cathodal | 1mA | NM | Anodal:F3; cathodal:SO | NM | 10 | NM | 15min |  |
| Barham et al.2022 | tDCS | Anodal/Cathodal | 2mA | Bi-DLPFC | Anodal:F4; cathodal:F3 | 5 times | 5 | 1 | 20min |  |
| Dakwar-Kawar et al.2022 | tDCS/tRNS | Anodal | 0.75mA | L-DLPFC | Anodal:F3; cathodal:Fp2/ Anodal: DLPFC; cathodal: IFG | 2w | 5 active+5 sham | 5/w | 20min |  |
| Kannen et al.2022 | tACS | NM | 1mA | NM | C1, C2, C5, C6 | 2 times | 2 | 1 | 20min |  |
| Klomjai et al.2022 | tDCS | Cathodal | 1.5mA | L-DLPFC | Anodal: Fp2; cathodal: F3 | 2w | 5 active+5 sham | 5/w | 20min |  |
| Leffa et al.2022 | tDCS | Anodal+ Cathodal | 2mA | Bi-DLPFC | Anodal: F4; cathodal: F3 | 4w | 28 | 7/w | 30min |  |
| Liang et al.2022 | rTMS | HF | 10Hz | R-DLPFC | NM | 12w | 60 | 5/w | NM |  |
| Makkar et al.2022 | tDCS | Anodal+Cathodal | 1mA | L-DLPFC/R-OFC | Anodal: F3; cathodal: Fp2 | 4w | 12 | 3/w | 20min |  |
| Nagy et al.2022 | rTMS | HF | 10Hz | R-DLPFC | F4 | 3w | 15 | 5/w | NM |  |
| Nejati et al.2022 | tDCS | Anodal | 1mA | Exp1：R-DLPFC Exp2：L-DLPFC | Exp1：Anodal:R-F4; cathodal: L-forearm Exp2：Anodal: L-F3; cathodal: R-forearm | 2 times | 1Exp1+1Exp2 | 1 | 20min |  |
| Salehinejad et al.2022 | tDCS | Anodal | 1.5mA | Bi-DLPFC | Anodal: F3 and F4; cathodal: contralateral shoulders | 2 times | 1 left+1 right | 1 | 15min |  |
| Westwood et al.2022 | tDCS | Anodal | 1mA | R-IFC | Anodal: F8; cathodal: Fp1 | 3w | 15 | 5/w | 20min |  |
| Nejati et al.2023 | tDCS | NM | 1.5mA | L-DLPFC R-VMPFC | Anodal: F3; cathodal: Fp2 Anodal: Fp2; cathodal: F3 Anodal: F3; cathodal: shoulder Anodal: Fp2; cathodal: shoulder | 5 times | 5 | 1 | 15min |  |
| Bian et al.2024 | rTMS | HF | 10 Hz | R-DLPFC | NM | 4w | 20 | 5/w | 30min |  |
| Cheung et al.2024 | TPS |  | 3 μs, 0.2-0.25 mJ/mm 2, 4-5 Hz/s | L-DLPFC | NM | 2w | 6 | 3/w | 30min |  |
| Estaji et al.2024 | tDCS | Anodal/Cathodal | 2mA | DLPFC/vmPFC | Anodal: F3 cathodal: Fp2; anodal: Fp2 cathodal: F3 | 3 times | 1Exp1+1Exp2+  1Exp3 | 1 | 20min |  |
| Guimarães et al.2024 | tDCS | Anodal | 2mA | L-DLPFC | Anodal: F3; Cathodal: Fp2 | 5 days | 5 | 1/d | 30min |  |
| Kannen et al.2024 | tACS | LF | 1.5mA | Cz,Oz | Cz ;Oz | 2 times | 1 active + 1 sham | 1/d | 18min |  |
| Nejati et al.2024 | tRNS | Anodal | 1mA | Bi-PFC | Anodal: F3;  Cathodal: FP2 | 2 times | 1 active + 1 sham | 1/w | 20min |  |
| Tian et al.2024 | rTMS | HF | 10 Hz | R-DLPFC | NM | 12w | 60 | 5/w | 20min |  |
| Wang et al.2024 | rTMS | LF | 1 Hz | Bi-DLPFC | NM | 12w | 36 | 3/w | 20min |  |
| Yang et al.2024 | rTMS | L-LF  R-HF | L-1 Hz  R-5 Hz | Bi-DLPFC | NM | 12w | 45 | every other day | NM |  |

EG: L:left; R:right; BI: bilateral; IFG：inferior frontal gyrus; DLPFC：dorsolateral prefrontal cortex area; HF: high-frequency; LF: low-frequency; dTMS：deep transcranial magnetic stimulation; PPC: posterior parietal cortex; TPS: transcranial pulse stimulation; tACS: transcranial alternating current stimulation

Table S7. Study design and measurement-related characteristics

| study | Study design | | Features of accompaning therapy | | | Comorbidity | Measurement-related characteristics | | Diagnostic criteria |
| --- | --- | --- | --- | --- | --- | --- | --- | --- | --- |
|  | Design type | Interval of  crossover | Type of accompaning  therapy | Duration of each  session | Is NIBS and  therapy concomitant | Comorbid psychiatric | Scale | Time of evaluation; follow-up |  |
| Cosmo et al.2015 | Double-blind RCT | NM | NM | NM | NM | NO | GNG task | Baseline; Immediately post | diagnoses by DSM-IV |
| Breitling et al.2016 | Crossover RCT | ≥1w | NM | NM | NM | CD/ ODD | Flanker task | Baseline; Immediately post | diagnoses by DSM-IV |
| Cachoeira et al.2017 | Double-blind RCT | NM | NM | NM | NM | NO | ASRS; SDS scores | baseline; end of treatment ;1,2 and 4 week after the last stimulation | diagnoses by DSM-V |
| Heng et al.2017 | RCT | NM | cognitive training | 60min | NM | NO | NCT; PSQ | baseline; end of treatment | diagnoses by DSM-IV |
| Soff et al.2017 | Double-blind crossover RCT | 2w | NM | NM | NM | NO | FBB-ADHD; Qb test | FBB-ADHD: baseline; end of treatment and 1 week after the last stimulation  Qb test: baseline; second to fifth day of stimulation and 1 week after the last stimulation | diagnoses by DSM-IV or for the hyperkinetic disorder according to ICD-10 |
| Allenby et al.2018 | Double-blind RCT | 2w | NM | NM | NM | NO | CPT; SSRT | baseline, end of treatment, and 3 days after the last stimulation | diagnoses by DSM-V |
| Cao et al.2018 | RCT | NM | ATX | 6w;7/w;1/d | yes | NO | SNAP-IV questionnaire; CPT; WISC test; IGT test | baseline; end of treatment | diagnoses by DSM-V |
| Paz et al.2018 | Double-blind RCT | NM | NM | NM | NM | NO | TOVA total score; CAARS | TOVA: baseline; tenth session; immediately after the last stimulation;1 week after treatment CAARS: baseline; immediately after the last stimulation | diagnoses by DSM-V and The Wender Utah ADHD Rating Scale and the Adult ADHD Self Report Scale |
| Soltaninejad et al.2019 | Crossover RCT | ≥72h | NM | NM | NM | NO | GNG task; Stroop task | Immediately post | diagnoses by CAARS-S:S and the Wender Utah Rating |
| Alyagon et al.2020 | Double-blind RCT | NM | NM | NM | NM | NO | CAARS; BAARS-IV; BRIEF-A; BDI; Stroop task | baseline; immediately after the last stimulation;1 month after the last treatment session | diagnoses by DSM-IV |
| Breitling et al.2020 | Double-blind pseudo-RCT | ≥6d | NM | NM | NM | CD/ ODD | n-back test | Immediately post | diagnoses by DSM-IV |
| Nejati et al.2020 | Double-blind crossover RCT | ≥72h | NM | NM | NM | NO | GNG task; Stroop task; N-back task; WCST tasks | Immediately post | diagnoses by Swanson, Nolan and SNAP-IV Parent Version Questionnaire |
| Salehinejad et al.2020 | Double-blind crossover RCT | ≥72h | NM | NM | NM | NO | Attention tasks; Stroop test; SAT; GNG task | Immediately post | diagnoses by DSM-V and their parents completed CPRS |
| Breitling-Ziegler et al.2021 | Double-blind crossover RCT | NM | NM | NM | NM | CD/ ODD | n-back task; GNG task; Flanker task; Spanboard task | baseline; immediately after the last stimulation; 19–23 weeks after the last treatment session | diagnoses by DSM- V |
| Westwood et al.2021 | Double-blind RCT | NM | cognitive training | 20min | yes | CD/ ODD | MARS; ADHD-RS; Mackworth Clock Task；WCST；Working Memory Task；GNG task；Simon；CPT；Conners 3-P ADHD Index；WREMB-R; CIS; ARI; MEWS | baseline, immediately after the last stimulation and 6-month after the last treatment session | diagnoses by DSM- V |
| Amouzadeh et al.2022 | Double-blind RCT | NM | NM | NM | NM | NO | Stroop test; N-Back test | baseline, immediately after the last stimulation and 2 weeks after the last treatment session | diagnoses by two psychiatrists |
| Barham et al.2022 | Double-blind RCT | NM | NM | NM | NM | NO | ASRS; DIVA.2.0; Edinburgh Handedness Inventory; Neuropsychological evaluation; Stroop Test; ToL task; CPT; RMET | baseline, immediately post | diagnoses by DSM- V, ASRS and DIVA 2.0 |
| Dakwar-Kawar et al.2022 | Double-blind crossover RCT | 1 w | Computerized Cognitive Training | 20min | yes | NM | MOXO-CPT | baseline; end of week 1; beginning week 3;end of week 3;beginning week 5 | diagnoses by DSM- V |
| Kannen et al.2022 | crossover RCT | NM | NM | NM | NM | NM | Visual Oddball Task; d2 Task | baseline, immediately post | already diagnosed with ADHD or were in the process of diagnosis at the specialized outpatient clinic for adult ADHD, and all participants underwent the IDA-R |
| Klomjai et al.2022 | Double-blind crossover RCT | ≥1month | NM | NM | NM | NO | Go/No-Go task; CPT | baseline, after 5 sessions, 1-week and 1-month after the last treatment session | diagnoses by DSM-V |
| Leffa et al.2022 | Double-blind RCT | ≥15h | NM | NM | NM | NO | CASRS-I; CASRS-HI; BDI; BRIEF-A; | Baseline; mid-treatment and post-treatment | diagnoses by DSM-V |
| Liang et al.2022 | RCT | NM | sensory integration training | 60min | no | NO | PSQ; SNAP-IV; IVA-CPT | baseline, immediately after the last stimulation | diagnoses by DSM-V |
| Makkar et al.2022 | unblinded RCT | NM | video game | 20min | yes | NO | RPM; Stroop test; TMT | Baseline; mid-treatment and post-treatment | diagnoses by parent and teacher versions of the NICHQ Vanderbilt assessment scale |
| Nagy et al.2022 | Double-blind RCT | NM | ATX | 1.2 mg / kg / day | yes | NO | Conner’s Parent Rating Scale-Revised-Long form; CGAS; CGI | Baseline; immediately after the last stimulation; 1-month after the last treatment session | diagnoses by DSM-Ⅳ |
| Nejati et al.2022 | single-blinded DDM modeling | ≥72h | NM | NM | NM | NO | GNG task；N- back test | Baseline; immediately after the last stimulation | diagnoses by DSM-Ⅴ |
| Salehinejad et al.2022 | crossover RCT | ≥72h | NM | NM | NM | NO | n-back test；WCST；GNG test; Flanker test | Baseline; immediately after the last stimulation | diagnoses by DSM-Ⅴ |
| Westwood et al.2022 | Double-blind RCT | NM | multi-executive functions training | 20min | yes | CD/ ODD | GNG Task | baseline, immediately after the last stimulation; 6-month after the last treatment session | diagnoses by DSM-Ⅴ and confirmed using K-SADS-PL |
| Nejati et al.2023 | Computational Modeling | ≥1w | NM | NM | NM | NO | CDDT；BART | Immediately post | diagnoses by DSM-Ⅴ |
| Bian et al.2024 | RCT | NM | methylphenidate | NM | yes | NO | Clinical efficacy; Conners Children Behavioral test; WISC-IV | baseline, immediately after the last stimulation | diagnoses by DSM-Ⅳ |
| Cheung et al.2024 | Double-blind RCT | NM | NM | NM | NM | NO | SNAP-IV, ADHD RS-IV, CGI; Stroop tests | Baseline; immediately after the last stimulation; 1-month and 3-month after the last treatment session | diagnoses by DSM-Ⅳ |
| Estaji et al.2024 | single-blinded RCT | 1 w | NM | NM | NM | NO | SNAP‑IV; ERC; BRIEF; GNG task;1‑back task | immediately after the last stimulation | diagnoses by DSM-Ⅳ |
| Guimarães et al.2024 | Double-blind crossover RCT | NM | NM | NM | NM | NM | TAVIS-4; Corsi Block-Tapping Task; Digit Span subtest; IR | baseline ;5 days after treatment | diagnoses by DSM-Ⅴ |
| Kannen et al.2024 | Blind crossover RCT | 1 day | NM | NM | NM | NM | 7-point Likert-scale; VSRQ | baseline, immediately after the last stimulation | administered the structured clinical “Interview of Integrated Diagnosis of ADHD in Adulthood” (IDA-R) |
| Nejati et al.2024 | crossover RCT | 1 w | NM | NM | NM | NO | circle tracing task; GNG task; 1-back task; WCST; BART | Immediately post | diagnoses by DSM-Ⅴ |
| Tian et al.2024 | Retrospective Study | NM | tiapride | 50 mg/time;2 times/day; 3 months | yes | NO | SNAP-IV scores; PSQ; WFIRS-P | baseline ;immediately after the last stimulation | met the diagnostic criteria of ADHD in children in the Diagnostic and Statistical Manual of Mental Disorders |
| Wang et al.2024 | RCT | NM | Behavioral Therapy; Applied Behavior Analysis; Attention Training: | 20min | yes | NO | SNAP-IV scale scores; Go/No-Go task; fNIRS | baseline; immediately after the last stimulation | diagnoses by DSM-Ⅳ |
| Yang et al.2024 | Controlled trial | NM | methylphenidate | 18 mg/time;1 times/day; 3 months | yes | NO | Conners Child Behavior Scale; Clinical Assessment of Attention Deficit; Clinical efficacy | baseline; immediately after the last stimulation | diagnoses by DSM-Ⅴ |

EG: CD: Conduct disorder; ODD: Oppositional defiant disorder; NCT: Number Cancel Test; PSQ: Parents Symptom Questionnaire; SSRT: stop signal reaction time; ATX: Atomoxetine; IGT: iowa gambling Tasks; TOVA: Test of Variables of Attention; CAARS: Conners’Adult ADHD Rating Scale; MARS: Maudsley Attention and Response Suppression; ADHD-RS: Parent-rated ADHD Rating Scale-IV; WREMB-R: Morning Behaviour-Revised scale; CIS: Columbia Impairment Scale-Parent version; ARI: child- and caregiver-rated Affective Reactivity Index; MEWS: child-rated Mind Excessively Wandering Scale; ASRS：Adult Self-Report Scale for ADHD; DIVA 2.0：Diagnostic Interview for ADHD in Adults 2.0 ; DSM-Ⅴ：the fifth edition of Diagnostic and Statistical Manual of Mental Disorders ; IDA-R: Interview of Integrated Diagnosis of ADHD in Adulthood; CASRS-I：Inattentive scores in the clinician-administered version of the Adult ADHD Self-report Scale version 1.1; SNAP-IV: Swanson, Nolan and Pelham-IV rating scales; CPT: continuous performance test; K-SADS-PL: Schedule of Affective Disorders and Schizophrenia for School-Age Children-Present and Lifetime version; CDDT: Chocolate delay discounting task; BART: Balloon analogue risk task; ERC: Emotion regulation checklist; BRIEF: Behavior rating inventory of executive function; TAVIS-4: Visual Attention Test, Fourth Edition; IR: Inhibiting Response subtest; VSRQ: Virtual Reality Sickness Questionnaire; WFIRS-P: Weiss Functional Impairment Rating Scale—Parent Report; CGI: Clinical global impression; WISC: Wechsler Intelligence Scale for Children; WCST: Wisconsin card sorting test; CGAS: Children’s Global Assessment Scale; RPM: Raven progressive matrices; TMT: trail making test; BDI: Beck Depression Inventory; RMET: Reading the Mind in the Eyes Test; SAT: Shifting attention test; BAARS-IV: Barkely Adult ADHD Rating Scale; SDS: Sheehan Disability Scale;
